# Telehealth-Delivered Dietary Counseling in Myeloproliferative Neoplasms: A Randomized Feasibility Study

**DOI:** 10.3390/nu18071158

**Published:** 2026-04-04

**Authors:** Angela Fleischman, Jiarui Li, Asmaa Tabban, Shuwei Cai, Andrew Odegaard

**Affiliations:** 1 Irvine School of Medicine, University of California, Irvine, CA 92617, USA; 2 Chao Family Comprehensive Cancer Center, University of California, Irvine, CA 92617, USA; 3 Irvine School of Public Health, University of California, Irvine, CA 92617, USAaodegaar@hs.uci.edu (A.O.)

**Keywords:** myeloproliferative neoplasms, Mediterranean diet, DASH diet, telehealth, dietary intervention, feasibility, symptom burden, diet quality, cardiovascular risk, patient-reported outcomes

## Abstract

**Background/Objectives:** Patients with myeloproliferative neoplasms (MPNs) experience chronic inflammation, elevated cardiovascular risk, and substantial symptom burden. Dietary patterns with anti-inflammatory and cardioprotective effects may represent a modifiable strategy to address these overlapping risks, yet dietary intervention has not been systematically studied in MPN. We evaluated the feasibility, engagement, and preliminary clinical signals of a fully remote dietary counseling intervention in adults with MPN. **Methods:** In this single-center, randomized, open-label pilot study, 28 adults with polycythemia vera, essential thrombocythemia, or primary myelofibrosis were randomized 1:1 to Mediterranean (MED) or Dietary Approaches to Stop Hypertension (DASH) dietary counseling over 10 weeks. The protocol included a 2-week baseline run-in period, 10-week active intervention with four telehealth dietitian visits, and 4-week postintervention follow-up. Prespecified feasibility endpoints were the completion of dietitian visits, daily MPN Symptom Assessment Form Total Symptom Score (MPN-SAF TSS) surveys, Mediterranean Diet Adherence Screener (MEDAS) questionnaires, and Automated Self-Administered 24-Hour Dietary Recall (ASA24) assessments. Exploratory endpoints included the change in Healthy Eating Index 2015 (HEI-2015) and symptom burden. **Results:** Twenty-seven participants provided data and were analyzed (14 MED, 13 DASH). Dietitian visit attendance was 96% (MED) and 85% (DASH). Daily symptom survey completion averaged 93% (MED) and 58% (DASH). MEDAS completion was 81% (MED) and 51% (DASH); ASA24 completion was 55% (MED) and 38% (DASH). HEI-2015 increased from 55 to 63 in MED during active intervention. At week 12, 23% of MED and 13% of DASH participants achieved ≥50% TSS reduction. Symptom reductions were observed across multiple domains. **Conclusions:** A fully remote dietary intervention is feasible in adults with MPN, with strong engagement in the Mediterranean arm. These findings support adequately powered trials incorporating biomarker endpoints to evaluate dietary modification as a strategy for inflammation-driven symptoms and cardiovascular risk in MPN.

## 1. Introduction

Polycythemia vera, essential thrombocythemia, and primary myelofibrosis are chronic myeloproliferative neoplasms (MPNs) situated at the intersection of malignancy, chronic inflammation, and cardiovascular disease. Although a diagnosis of MPN is rare, affecting approximately 5 in 100,000 people [1], the presence of the JAK2V617F mutation, the most common MPN driver mutation, is substantially more common, reaching a population prevalence as high as 3.1% when detected with highly sensitive methods [2]. Thrombosis is a leading cause of morbidity and mortality, involving both arterial and venous events [3,4,5,6,7,8]. The JAK2V617F mutation drives constitutive JAK-STAT signaling, amplifying systemic inflammation and accelerating atherosclerosis in mouse models in a cholesterol-dependent manner, with aggressive LDL lowering shown to normalize plaque regression even in the setting of JAK2V617F clonal hematopoiesis [9]. Treatment in MPN is focused on the reduction of thrombotic risk, management of blood counts, and symptom control. Cytoreductive agents such as hydroxyurea, JAK inhibitors, and interferon-alpha are reserved for higher-risk patients, while many lower-risk patients are managed with aspirin alone. The pathogenesis is multifactorial [10,11], with contributions from activated platelets [12], endothelial cell dysfunction [13], and neutrophil extracellular traps [14]. Adults with MPN have an elevated risk of atherothrombotic events compared with the general population [7,8,15,16], and cardiovascular risk reduction is a primary objective of current treatment guidelines [17]. However, these guidelines do not specify strategies to achieve this goal, and lifestyle interventions remain underutilized in clinical practice.

Dietary modification is foundational to cardiovascular disease prevention. The Mediterranean dietary pattern reduces major adverse cardiovascular events and lowers inflammatory biomarkers in high-risk individuals [18,19]. The DASH dietary pattern improves blood pressure, lipid profiles, oxidative stress, and systemic inflammation [20,21,22,23,24,25]. Both patterns emphasize fruits, vegetables, whole grains, and lean proteins while limiting processed foods and added sugars, but they differ in specific recommendations: the Mediterranean pattern emphasizes olive oil as a primary fat source, moderate wine consumption, and liberal use of nuts and legumes, whereas DASH specifically targets sodium reduction and emphasizes low-fat dairy intake. The pathways targeted by these diets, including endothelial function, oxidative stress, lipid metabolism, and inflammation, overlap directly with mechanisms implicated in MPN-associated thrombosis. Diet therefore represents a biologically plausible strategy to modify vascular risk in MPN.

Inflammation in MPN contributes not only to thrombosis but also to symptom burden. Cytokine-driven signaling is associated with fatigue, pruritus, and constitutional symptoms [26]. An intervention that attenuates systemic inflammation may therefore influence both cardiovascular risk and patient-reported outcomes, addressing multiple clinically meaningful endpoints simultaneously.

Despite this rationale, dietary intervention has not been systematically evaluated in MPN. In a prior pilot study, we demonstrated that adults with MPN can adopt a Mediterranean dietary pattern with structured in-person counseling [27]. However, in-person delivery limits scalability and access, and dietary counseling is not currently integrated into routine MPN care despite the guideline emphasis on cardiovascular risk management. Telehealth models offer an opportunity to deliver dietitian-led counseling within longitudinal MPN care without expanding clinic burden.

The present study was designed to evaluate the feasibility and engagement of a fully remote dietary intervention delivered via telehealth in adults with MPN. Participants were randomized to Mediterranean or DASH dietary counseling and completed daily electronic symptom surveys over 16 weeks. The primary objectives were feasibility and engagement with study activities. Secondary exploratory objectives included changes in diet quality and longitudinal symptom trajectories.

## 2. Materials and Methods

### 2.1. Study Design and Participants

This single-center, randomized, open-label pilot study tested the feasibility of a fully remote dietary intervention for adults with MPN at the University of California, Irvine. The study was conducted from February through September 2021 with IRB approval. The protocol was approved by the UCI Institutional Review Board and registered at ClinicalTrials.gov (NCT04744974, 8 February 2021). Adults with a confirmed diagnosis of polycythemia vera, essential thrombocythemia, or primary myelofibrosis were eligible. Inclusion and exclusion criteria are shown in Table 1.

### 2.2. Recruitment, Run-In Period/Baseline and Randomization

Participants were recruited through passive advertisement at MPN patient events and online patient communities. Participants completed an enrollment intake survey of dietary habits and symptom burden (survey included in Supplementary Materials). After enrollment, their baseline symptom burden and diet quality were assessed with daily symptom surveys and three 24 h diet recalls. Participants were randomized 1:1 to either the MED or DASH dietary pattern using simple randomization implemented 24 h before the first dietitian visit.

### 2.3. Outcome Measures

The primary objective was to evaluate the feasibility of the fully remote intervention model. Four prespecified feasibility endpoints were assessed: (1) the proportion of scheduled dietitian visits attended (four per participant), (2) the completion rate of daily symptom surveys (112 total over 16 weeks), (3) the completion rate of Mediterranean Diet Adherence Screener (MEDAS) questionnaires administered at weeks 2, 8, and 14, and (4) the completion rate of Automated Self-Administered 24-Hour Dietary Recall (ASA24) assessments collected during the preintervention period and at unannounced intervals during weeks 7, 8, 13, 14, and 15. The background and rationale for each of these tools is detailed in Supplementary Table S1. All surveys were prompted by email, and participants self-completed all assessments without staff assistance.

Secondary endpoints included change in diet quality assessed by the Healthy Eating Index 2015 (HEI-2015) [28] derived from ASA24 recall data and change in symptom burden measured by the MPN-SAF TSS (MPN-10) [26], a validated 10-item patient-reported outcome instrument scored from 0 to 100, with higher scores indicating greater symptom burden.

### 2.4. Intervention

**Figure 1:**
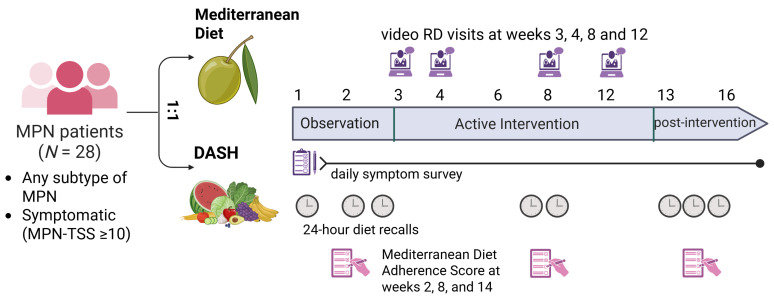
MPN E-nutrition intervention study design.

The total study duration was 16 weeks (Figure 1). Weeks 1 through 2 constituted a preintervention baseline period during which participants completed daily symptom surveys and three unannounced 24 h dietary recalls without receiving dietary counseling. The active intervention spanned weeks 3 through 12. At randomization, participants received a written curriculum on their assigned dietary pattern (Educational Materials used included in Supplementary Materials). Four individualized video visits with a registered dietitian were scheduled at weeks 3, 4, 8, and 12. Each visit followed a standardized structure: the dietitian reviewed the previous week, assessed barriers, provided tailored education on the assigned pattern, established one to two behavioral goals, and discussed strategies for shopping and meal preparation. Visits lasted approximately 30 to 45 min. Dietitian scripts for each visit are included in Supplementary Materials. Between visits, participants received email reminders and had continued access to the written curriculum and sample recipes. Weeks 13 through 16 constituted a postintervention observation period in which participants no longer received dietitian counseling but continued to be monitored with daily symptom surveys (MPN-SAF), three unannounced 24 h diet recalls (ASA-24) and one MEDAS, all obtained via email prompts.

### 2.5. Statistical Analysis

This study was designed to assess feasibility and was not powered for hypothesis testing of between-group differences. Descriptive statistics were used throughout. Continuous variables are presented as means with standard deviations (SD) and categorical variables as counts with percentages. Each feasibility endpoint was calculated as the number of completed assessments divided by the number scheduled, then averaged within each dietary arm. Participants who withdrew were included in feasibility denominators based on scheduled assessments.

HEI-2015 scores were calculated from ASA24 data using published scoring algorithms and averaged within each study period: P1 (weeks 1–2), P2 (weeks 3–12), and P3 (weeks 13–16). Daily TSS scores were averaged by week and by study period using the same intervals. Because an early decline in TSS was observed during week 1 in multiple participants prior to any dietitian contact, week 2 average TSS was used as the operational baseline for all pre-post comparisons. A clinically meaningful response was defined as at least 50% reduction in TSS from baseline to week 12. Given the pilot nature and small sample size, no formal between-group statistical comparisons were performed. Analyses were conducted using GraphPad Prism 10.6.1.

### 2.6. Use of Generative AI

Claude AI was used for grammatically refinement of the manuscript. AI was not used to interpret data or generate figures.

## 3. Results

### 3.1. Cohort and Baseline Characteristics

Twenty-eight adults with MPN were enrolled and randomized, 14 to the MED arm and 14 to the DASH arm. One participant randomized to DASH (E15) withdrew prior to the first dietitian counseling session and completed no study assessments; this participant was excluded from analyses. Three additional DASH participants (E4, E9, E14) attended at least one dietitian visit and contributed partial data but did not complete the full study protocol. No participants in the MED arm withdrew. The analytic cohort therefore comprised 27 participants (14 MED, 13 DASH), including three DASH participants with partial data.

Baseline demographic and clinical characteristics are shown in Table 2. The cohort was predominantly female (MED 79%, DASH 69%), White (MED 79%, DASH 85%), and non-Hispanic (MED 93%, DASH 100%). Mean age was 57.8 years (SD 8.4) in MED and 54.2 years (SD 14.8) in DASH.

Baseline symptom burden was moderate in both arms. Mean MPN-SAF TSS during the baseline period (weeks 1–2) was 17.7 (SD 8.6) in MED and 21.5 (SD 12.1) in DASH. Baseline diet quality assessed by HEI-2015 derived from ASA24 recalls was similar between groups (MED 54.6, SD 12.8, *n* = 13; DASH 59.0, SD 17.5, *n* = 12).

### 3.2. Feasibility: Engagement with Study Activities

**Figure 2:**
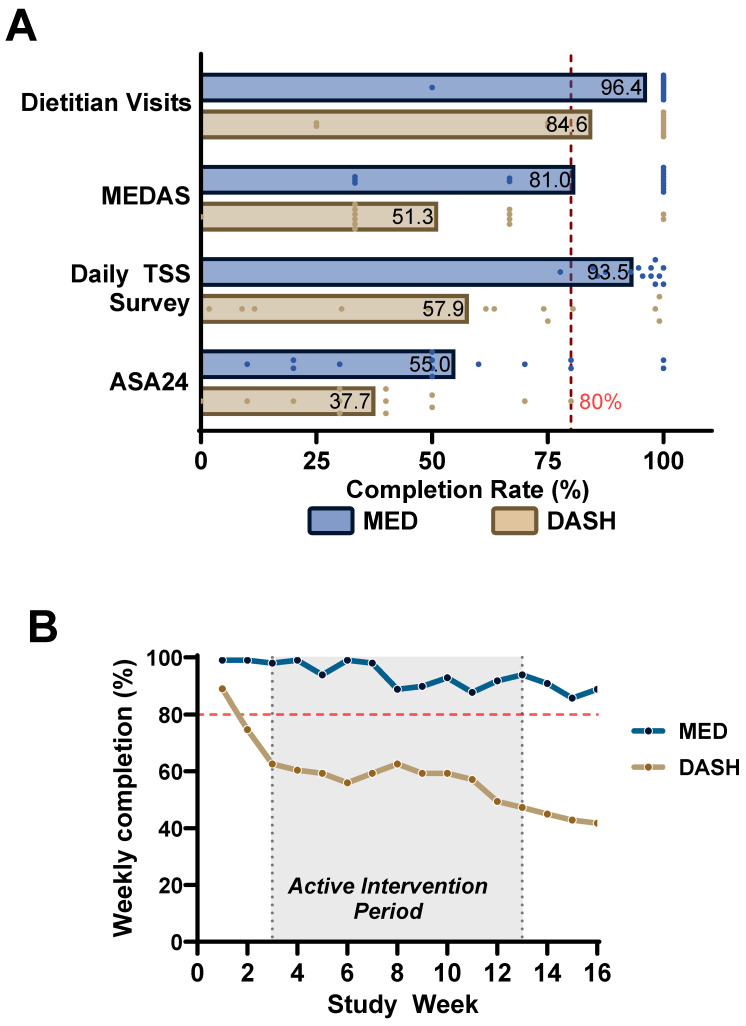
Engagement with study activities. (**A**) Mean completion rates for four prespecified feasibility metrics by diet arm. Dashed red line indicates the 80% feasibility threshold. (**B**) Weekly completion rate of daily MPN-SAF TSS surveys. Thick lines represent group means. Red dotted line represents the predefined goal of 80%. Grey area represents active intervention period.

Engagement was assessed using four prespecified feasibility metrics (Figure 2A). Dietitian visit attendance was the highest across metrics, with mean completion rates of 96% in MED (54/56 visits) and 85% in DASH (44/52 visits). In the DASH arm, attendance declined from 100% at visit 1 to 69% at visit 4; in MED, attendance remained ≥93% at all visits.

Daily MPN-SAF TSS survey completion averaged 93% in MED and 58% in DASH across the 16-week study. MEDAS questionnaire completion was 81% in MED and 51% in DASH. ASA24 dietary recall completion was 55% in MED and 38% in DASH. Across all four metrics, completion rates were consistently higher in the MED arm and decreased in proportion to assessment burden.

Weekly completion rates for daily MPN-SAF TSS surveys are shown in Figure 2B. In MED, weekly completion remained above 86% throughout the study and was 89% at week 16. In DASH, completion declined from 89% in week 1 to 42% at week 16.

### 3.3. Diet Quality Changes

**Figure 3:**
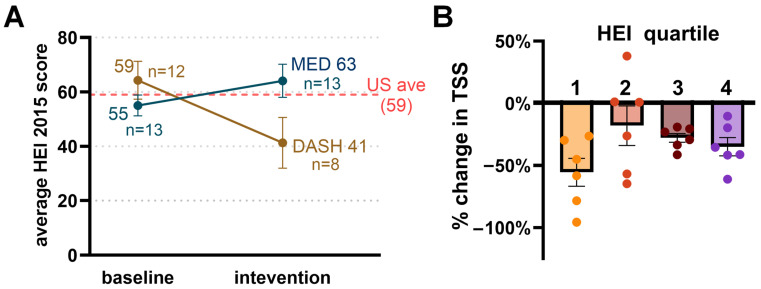
Changes in Healthy Eating Index and change in symptom burden. (**A**). Average HEI 2015 at baseline (weeks 1–2) versus during active intervention (weeks 3–12) comparing MED and DASH groups (**B**). Percent change in TSS during active intervention versus baseline of entire cohort separating group into HEI quartile at baseline (Quartile 1 being lowest HEI).

Diet quality was assessed using the HEI-2015 calculated from ASA24 recall data. At baseline, mean HEI-2015 scores were 55 (SD 13, *n* = 13) in the MED arm and 59 (SD 18, *n* = 12) in the DASH arm (Figure 3A). During active intervention, mean HEI-2015 increased in the MED group from 55 to 63 (*n* = 13), whereas HEI-2015 declined in the DASH group from 59 to 41 (*n* = 8). Among participants with paired baseline and intervention recalls, 9 of 12 (75%) in the MED arm showed individual improvement, compared with 3 of 8 (38%) in the DASH arm.

To explore whether baseline diet quality modified symptom response, participants from both arms were pooled and stratified by baseline HEI-2015 quartile (Figure 3B). Those in the lowest baseline HEI quartile showed the largest mean symptom reductions (−56%, *n* = 6), while the second quartile showed the smallest (−18%, *n* = 6). The third and fourth quartiles showed intermediate reductions (−28% and −35%, respectively).

### 3.4. Symptom Burden Changes

An early decline in TSS was observed during week 1 in multiple participants, prior to any dietitian contact or dietary instruction. Mean TSS at week 2 was, therefore, designated as the operational baseline for all pre-post comparisons.

**Figure 4:**
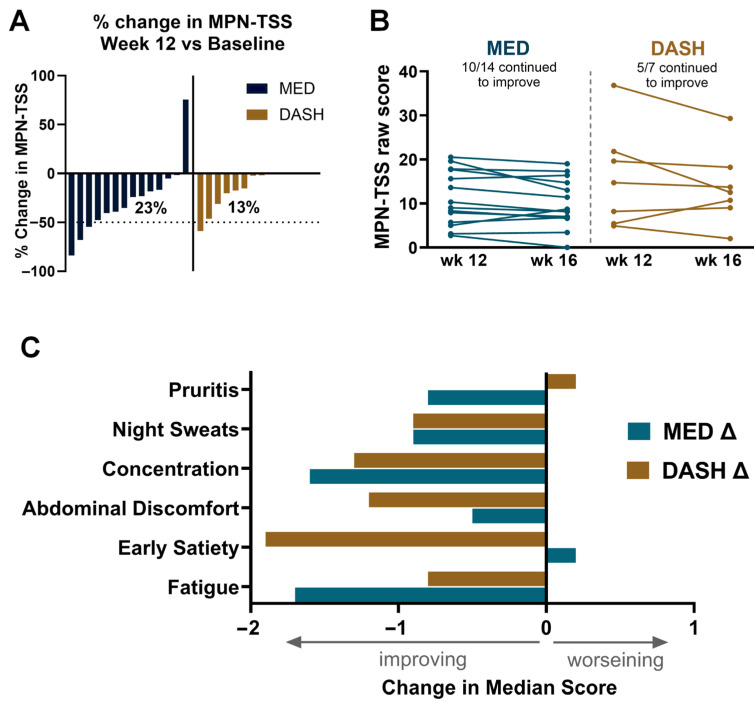
Change in symptoms with diet intervention. (**A**) Percent change in mean MPN-TSS at week 12 (end of intervention period) versus baseline (defined as week 2), each bar on x axis represents a single participant. (**B**) Raw change in mean MPN-TSS at week 16 (end of follow-up period) versus week 12. Only participants with at least five completed surveys during week 12 and week 2 are included. (**C**) Change in each symptom week 12 versus baseline. Median raw score at week 12 versus week 2 is shown.

Among participants with adequate survey data at both time points, 3 of 13 MED participants (23%) and 1 of 8 DASH participants (13%) achieved at least a 50% reduction in TSS from baseline to week 12 (Figure 4A). The majority of participants in both arms showed some degree of symptom reduction, with most experiencing moderate improvement below the 50% threshold.

Symptom improvement was sustained in many participants beyond the active counseling period. During weeks 13 through 16, 10 of 14 MED participants (71%) and 5 of 7 DASH participants (71%) showed further TSS decline at week 16 compared with week 12 (Figure 4B).

Symptom domain analysis revealed reductions across multiple MPN-SAF domains, including fatigue, early satiety, abdominal discomfort, concentration difficulties, night sweats, pruritus, and bone pain (Figure 4C). These decreases were observed in both arms and were not confined to a single symptom cluster.

### 3.5. Longitudinal Symptom Trajectories

**Figure 5:**
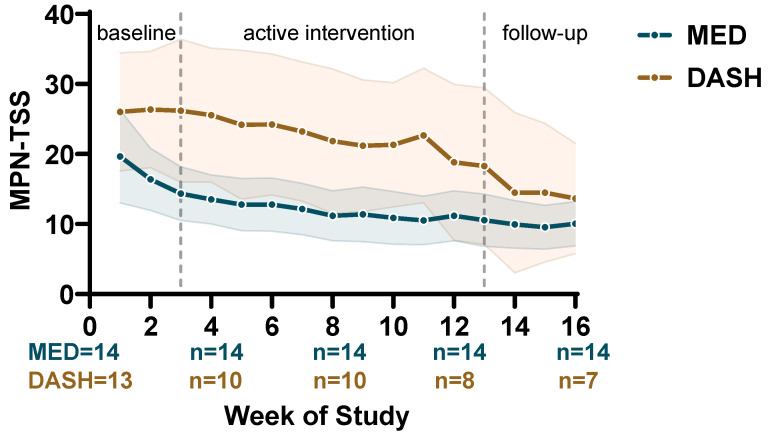
Longitudinal MPN-SAF TSS trajectories by diet arm. Weekly mean Total Symptom Score (MPN-SAF TSS) is plotted with 95% confidence intervals (shaded bands) for the Mediterranean (MED, blue) and DASH (gold) arms across the 16-week study. Vertical dashed lines delineate study phases: baseline observation (weeks 1–2), active dietary intervention with telehealth dietitian counseling (weeks 3–12), and post-intervention follow-up (weeks 13–16). All 14 MED participants contributed data throughout the study; DASH participants declined from 13 (week 1) to 7 (week 16). The widening of the DASH confidence interval reflects this progressive attrition. Baseline is defined as week 2 average TSS.

The daily symptom monitoring protocol generated over 2300 individual TSS observations across the 16-week study, providing sufficient granularity to examine within-arm trajectories at weekly resolution (Figure 5). In the MED arm, mean TSS declined from 16.4 at baseline (week 2) to 11.2 at week 12, a reduction of 5.2 points (31.7%), and continued to decline to 10.1 at week 16. All 14 MED participants contributed data throughout the study, and the confidence interval remained narrow across all three phases. In the DASH arm, mean TSS declined from 26.3 to 18.8 at week 12 (7.5 points, 28.6%), with an apparent further decline to 13.6 at week 16. However, the number of DASH participants contributing data decreased from 12 at baseline to 7 by week 16.

Both arms showed gradual, progressive symptom reduction over the course of the active intervention rather than an abrupt change at any single time point. No formal between-arm comparison was performed given the baseline imbalance in TSS, differential attrition, and the pilot design.

## 4. Discussion

Adults with myeloproliferative neoplasms face two interrelated and persistent clinical challenges: chronic systemic inflammation and markedly elevated cardiovascular risk. Thrombosis remains a leading cause of morbidity cytoreductive therapy, and current NCCN guidelines list management of cardiovascular risk factors as a primary treatment objective [17]. Yet dietary counseling, which is foundational to cardiovascular risk reduction in other high-risk populations, is not embedded in routine MPN care. This disconnect reflects the structural realities of care delivery. MPN patients are primarily managed by oncologists whose clinical priorities appropriately center on hematologic control, thrombosis prevention, and mitigation of transformation risk. Counseling regarding dietary strategies to reduce cardiovascular risk and inflammation rarely fits within the time constraints of oncology visits. At the same time, primary care physicians and cardiologists may defer disease-specific guidance, viewing MPN as a malignant condition outside traditional cardiovascular management paradigms. As a result, although cardiovascular risk modification is formally prioritized, there is no clear ownership of dietary intervention in this population.

This gap has important implications. MPN is characterized by constitutive JAK-STAT activation, myeloid-driven inflammation, endothelial dysfunction, platelet hyperreactivity, and accelerated atherosclerosis [10,11,12,13,14,15,16]. Adults with this biology may require dietary guidance that is tailored to their distinct prothrombotic and proinflammatory physiology rather than extrapolated from the general population alone. Moreover, because dietary counseling does not naturally reside within oncology, cardiology, or primary care, delivering such guidance requires a dedicated model. These realities create a compelling opportunity for centralized, telehealth-delivered nutritional counseling that is disease-informed, scalable, and integrated into specialty care without expanding clinic burden.

Within this context, the present study demonstrates that a fully remote, dietitian-led dietary intervention is feasible in adults with MPN. Telehealth visit attendance approached 100% among participants who remained in the study, and daily symptom survey completion was sustained at 94% in the MED arm over 16 weeks. Engagement patterns revealed a clear inverse relationship between assessment burden and adherence: ASA24 dietary recalls, which require 30 to 60 min per session, had the lowest completion rates (52% MED, 40% DASH), whereas brief daily surveys requiring approximately two minutes were completed reliably. These findings directly inform future trial design and suggest that self-administered 24 h dietary recalls should be supplemented or replaced by staff-administered phone-based recalls to improve data capture without increasing participant burden.

Diet quality improved in the MED arm, with a mean increase in HEI-2015 from 55 to 63 during active intervention, and 75% of MED participants with paired recalls showed individual improvement. HEI-2015 did not improve in the DASH arm among participants who completed follow-up recalls, though interpretation is limited by the smaller number of completed recalls during intervention (*n* = 8), and the apparent decline may partly reflect selective attrition. The structural differences between the two patterns may be relevant. DASH includes explicit sodium reduction targets and structured guidance around low-fat dairy and portion balance, whereas the Mediterranean pattern emphasizes substitution (replacing saturated fats with olive oil, increasing legumes, nuts, and whole grains) without emphasizing restriction. These differences may influence perceived flexibility and long-term adherence. Because modulation of inflammatory and vascular pathways likely requires sustained dietary exposure, patterns that are easier to maintain over time may ultimately have greater biological relevance in MPN.

Symptom burden declined in both arms, with 23% of MED and 13% of DASH participants achieving at least 50% reduction in MPN-SAF TSS by week 12. Reductions were observed across multiple symptom domains, including fatigue, night sweats, pruritus, abdominal discomfort, and cognitive symptoms. The daily monitoring protocol, which generated over 2300 individual observations, revealed that symptom improvement followed a gradual, progressive trajectory rather than an abrupt change at any single time point, consistent with the biological expectation that anti-inflammatory dietary effects accrue over weeks. This granularity would not have been captured by the single time-point assessments used in most MPN clinical trials. Although this study did not include biomarker endpoints, the symptom trajectories are consistent with a plausible anti-inflammatory effect of dietary modification and support mechanistic evaluation in future trials.

Participants in the lowest baseline diet quality quartile showed the largest mean symptom reductions (−54%), suggesting that those with the most room for dietary improvement may derive the greatest symptomatic benefit. If confirmed in larger studies, baseline diet quality could serve as an enrichment criterion for selecting patients most likely to respond to nutritional intervention. This observation also has implications for study design: enrolling participants with uniformly high baseline diet quality would likely attenuate any treatment effect.

This study has limitations. It was not powered for between-arm efficacy comparisons, baseline symptom burden differed numerically between groups (MPN-SAF TSS 16.4 vs 26.3), and differential attrition in the DASH arm complicates interpretation of later time points. The cohort was predominantly White, non-Hispanic, and college-educated, reflecting the self-referral recruitment strategy and limiting generalizability. Increasing diversity in future trials will require proactive community outreach and culturally inclusive dietary framing; the term “Mediterranean diet” itself may limit perceived relevance among some populations, and alternative descriptions such as “plant-forward” or “heart-healthy” could broaden appeal. Dietary intake was assessed using self-administered recalls with modest completion rates, and the absence of biomarker data precludes mechanistic conclusions. All assessments were self-administered without staff oversight, and independent verification of responses was not possible. The differential engagement patterns between arms and the time-varying decline in DASH completion are consistent with authentic participation, though feasibility estimates should be interpreted within the context of this specific, highly motivated patient population. 

## 5. Conclusions

This pilot establishes that centralized, telehealth-delivered dietary counseling in a rare hematologic malignancy population is feasible and well accepted. Given the central role of inflammation and vascular dysfunction in MPN pathobiology, dietary intervention should now move from feasibility testing to adequately powered, biomarker-integrated randomized trials designed to determine whether nutritional modulation can alter disease-relevant cardiovascular and inflammatory endpoints. Future trials should incorporate serum biomarker endpoints, including inflammatory cytokines and markers of cardiovascular risk, to determine whether the dietary changes observed here translate into measurable biological effects.

## Figures and Tables

**Table 1:** Inclusion and Exclusion Criteria.

**Inclusion Criteria** Age ≥ 18 with a diagnosis of a Philadelphia chromosome negative MPN including ET, PV, or MF Has access to the internet and email MPN-TSS score of ≥10 on screening survey Mediterranean Adherence score of ≤10 on screening survey English fluency (intervention requires conversations with study staff) In the opinion of the study team is amenable to changing one’s diet
**Exclusion Criteria** Pregnant or planning to become pregnant over the course of the study Has food allergies, intolerances, or other dietary restrictions which would severely limit changes to their diet toward a Mediterranean style diet (such as allergies to ALL tree nuts or olive oil)

**Table 2:** Baseline demographic and clinical characteristics of study participants.

	DASH (*n* = 13) ^†^	MED (*n* = 14)
Age, mean (SD), y	54.2 (14.8)	57.8 (8.4)
Range	26–72	42–69
Gender, *n* (%)		
Female	9 (69.2)	11 (78.6)
Male	4 (30.8)	2 (14.3)
Other	—	1 (7.1)
Race, *n* (%)		
White	11 (84.6)	11 (78.6)
Black or African American	1 (7.7)	—
Other/Multiple	1 (7.7)	2 (14.3)
Prefer not to say	—	1 (7.1)
Ethnicity, *n* (%)		
Not Hispanic or Latino	13 (100)	13 (92.9)
Hispanic or Latino	—	1 (7.1)
Education, *n* (%) ‡		
College graduate or above	7 (70.0)	10 (71.4)
Some college or AA degree	2 (20.0)	3 (21.4)
High school graduate/GED	—	1 (7.1)
Other	1 (10.0)	—
Marital status, *n* (%) ‡		
Married or living with partner	7 (70.0)	10 (71.4)
Divorced or widowed	2 (20.0)	2 (14.3)
Never married	1 (10.0)	2 (14.3)
Household income, *n* (%) ‡		
≥$100,000	4 (40.0)	7 (50.0)
$15,000–$99,999	3 (30.0)	5 (35.7)
Prefer not to say	3 (30.0)	2 (14.3)
Prescription medication use, *n* (%) ‡		
Yes	8 (80.0)	12 (92.3)
No	—	1 (7.7)
Prefer not to answer	2 (20.0)	—
Baseline MPN-SAF TSS, mean (SD) §	21.5 (12.1)	17.7 (8.6)
Baseline HEI-2015, mean (SD) ‖	59.0 (17.5)	54.6 (12.8)

† One DASH participant (E15) withdrew before the first counseling session and completed no study assessments; this participant was excluded from all analyses. Three DASH participants (E4, E9, E14) are classified as dropouts but attended at least one dietitian visit and are retained in the analytic cohort. ‡ Three DASH participants (E13, E14, E28) did not complete the baseline questionnaire. Education, marital status, income, and medication percentages for the DASH arm are based on *n* = 10 with available data. § MPN-SAF TSS = MPN Symptom Assessment Form Total Symptom Score. Baseline values reflect average daily TSS during weeks 1–2. DASH *n* = 13, MED *n* = 14. ‖ HEI-2015 = Healthy Eating Index 2015, derived from ASA24 dietary recalls during weeks 1–2. DASH *n* = 12, MED *n* = 13. Values are *n* (%) unless otherwise specified. “—” indicates no participants in that category.

## Data Availability

The raw data supporting the conclusions of this article will be made available by the authors on request due to the nature of the data.

## Supplementary Materials

The following supporting information can be downloaded at https://www.mdpi.com/article/10.3390/nu18071158/s1, Table S1. Outcome Measures and Assessment Tools.

## Abbreviations

The following abbreviations are used in this manuscript:

AA	Associate of Arts
ASA24	Automated Self-Administered 24-hour Dietary Recall
CI	confidence interval
DASH	Dietary Approaches to Stop Hypertension
GED	General Educational Development
HEI-2015	Healthy Eating Index 2015
IRB	Institutional Review Board
JAK-STAT	Janus kinase-signal transducer and activator of transcription
MED	Mediterranean
MEDAS	Mediterranean Diet Adherence Screener
MPN	myeloproliferative neoplasm
MPN-SAF TSS	MPN Symptom Assessment Form Total Symptom Score
NCCN	National Comprehensive Cancer Network
RD	registered dietitian
SD	standard deviation
TSS	Total Symptom Score
