# Supplementary material for: Telehealth-Delivered Dietary Counseling in Myeloproliferative Neoplasms: A Randomized Feasibility Study
Source: Nutrients. 2026 Apr 4;18(7):1158. doi: 10.3390/nu18071158 (PMC13074705; doi:10.3390/nu18071158)
Supplement: Supplementary file 1 [file nutrients-18-01158-s001.zip › nutrients-4215737-supplementary.pdf]

Supplemental Materials Table of Contents

Table S1. Outcome Measures and Assessment Tools………………….Page 2

Dietitian Counseling Session Scripts……………………………………..Page 3

Enrollment Survey……………………………………………………….Page 4-12

DASH Diet Educational Materials……………………………………...Page 13-33

Mediterranean Diet Educational Materials………………………………Page 34-60

Supplementary Table S1. Outcome Measures and Assessment Tools

Survey / Tool	What It Measures	Rationale for Use	Key References
**MPN-SAF TSS** *(MPN-10)*	10-item patient-reported instrument scoring 10 MPN symptoms (fatigue, early satiety, abdominal discomfort, inactivity, concentration, night sweats, pruritus, bone pain, fever, weight loss) each from 0 to 10. Items summed for a total of 0 to 100; higher scores indicate greater burden. Administered daily over 16 weeks.	Standard patient-reported outcome instrument across MPN clinical trials, enabling direct comparison with prior literature including the NUTRIENT pilot. Daily administration captures symptom trajectory across baseline, active intervention, and post-intervention periods.	Scherber R, et al. Blood. 2011;118(2):401-408.<br>Emanuel RM, et al. J Clin Oncol. 2012;30(33):4098-4103.
**MEDAS** *(14-item)*	14-item PREDIMED consortium screener assessing Mediterranean dietary pattern adherence. Each item scores 0 or 1 based on consumption of key foods. Total score 0 to 14; a score of 8 or above indicates high adherence.	Standard adherence measure for Mediterranean diet trials; used in the prior NUTRIENT MPN study enabling direct comparison. Brief and self-administered, well-suited to repeated remote use with low participant burden. Served as the primary feasibility metric for dietary adherence in the MED arm.	Schroeder H, et al. J Nutr. 2011;141(6):1140-1145.<br>Mendez Luque LF, et al. Cancer Res Commun. 2024;4(3):660-670.
**ASA24** *(24-hr recall)*	NCI-developed web-based tool capturing detailed 24-hour dietary intake via automated multiple-pass method. Outputs include nutrient composition, food group servings, and dietary indices including HEI-2015. Administered at unannounced intervals to reduce reactivity.	Provides objective, interviewer-independent dietary intake data at low cost, ideal for remote administration. Unannounced timing reduces likelihood that participants alter intake in anticipation of assessment. Served as the data source for HEI-2015 calculation, the primary secondary endpoint for diet quality change.	Subar AF, et al. J Acad Nutr Diet. 2012;112(8):1134-1137.
**HEI-2015** *(Healthy Eating Index)*	Composite diet quality score measuring alignment with the 2015-2020 Dietary Guidelines for Americans across 13 components (9 adequacy, 4 moderation). Scores range from 0 to 100; higher scores indicate better overall diet quality. Calculated from ASA24 data using published NCI algorithms.	Captures overall diet quality rather than adherence to a single dietary pattern, enabling a common quantitative comparison of diet quality change across both the MED and DASH arms. Both dietary patterns should improve HEI-2015 scores, making it a useful arm-neutral secondary endpoint.	Krebs-Smith SM, et al. J Acad Nutr Diet. 2018;118(9):1591-1602.<br>Reedy J, et al. J Acad Nutr Diet. 2018;118(9):1622-1633.

Abbreviations: MPN-SAF TSS, Myeloproliferative Neoplasm Symptom Assessment Form Total Symptom Score; MEDAS, Mediterranean Diet Adherence Screener; ASA24, Automated Self-Administered 24-Hour Dietary Recall; HEI-2015, Healthy Eating Index 2015; NCI, National Cancer Institute; PREDIMED, Prevención con Dieta Mediterránea.

Initial counseling session

The initial counseling session is designed to familiarize the subject with the counselor, in this case the study Registered Dietitian. The subjects will be given an "Initial counseling form", which acts both as a document to the subject to outline his or her thoughts and as a structural guide to drive discussion during the session. The form asks about medical history, allergies and intolerances, supplements, medications, goals, work and stress, food relationships, dietary knowledge, concerns and readiness to change. Finally, the form will include a section on defining a SMART goal that will guide the subject on the proposed intervention until the next follow-up phone-call.

A SMART goal is an acronym for **S**pecific, **M**easurable, **A**chievable, **R**elevant and **T**imely goals. They are focused on a particular behavior, easy to quantify, realistic, worthwhile and trackable. Using them can provide a fundamental way to measure the subject's progression and help fine tune any obstacles along the way.

Follow-Up counseling sessions (phone-calls)

The follow-up sessions will mainly follow up on the challenges, behaviors and thoughts the subject encountered since their last session. It will specifically take a problem-solving approach to identify potential positives and negatives that the subject have experienced. The SMART goals will then be modified to reflect the best forward approach in order to mitigate potential obstacles.

Format

The initial session can be thought of as having 3 main parts. The first part introduces the study subject to the study dietitian and will aim to establish rapport. The second component will focus on discussing the subject responses to the "initial counseling form" and provide basic educational services on topics that will enhance the participant's knowledge of the intervention's aims and approaches. This may include discussing the nutritional science aspect of the proposed dietary changes along with troubleshooting potentially confusing topics at hand. The last component will seek to address the concerns and problems portion of the form and identify a SMART goal that the subject will aim to follow.

The follow-up sessions (phone calls) will be more specific and take a two-part approach. The first component will address concerns and problems the subject may have faced since the last session. The second component will aim to modify the SMART goal in order to better accommodate these changes if necessary.

Default Question Block

Thank you for your interest in an online intervention to test the impact on symptom burden in MPN patients.

This is a study being performed at UC Irvine under the direction of Dr. Angela Fleischman in the Department of Medicine. It is a 20 week study that involves online sessions, daily symptom surveys, and two saliva samples. All research activities can be performed in your home. You will not receive any compensation for this study.

To identify whether this study is right for you we are going to ask you some questions. Your answers will remain confidential and will only be used by our study team to determine your candidacy for this study. If you have questions about this study you may call 949-824-4144.

- ○ Yes, I understand and would like to proceed with the survey
- ○ No, I don't want to proceed with the survey
- ○ I'd like some more information before filling out the survey

We'd be happy to give you more information on this study. Please enter your preferred contact information below (email or phone), also describe any specific questions you have. If you prefer you can also email us at wearempn@gmail.com

Thank you for your interest. If you would like more information feel free to email us at wearempn@gmail.com. Also, if you have any comments you'd like to tell us please enter them below.

Block 6

Are you at least 18 years old?

- ○ Yes
- ○ No

Do you have a myeloproliferative neoplasm (MPN)? This includes:
Essential Thrombocythemia (ET)
Polycythemia Vera (PV)
Myelofibrosis (MF)

- ○ Yes
- ○ No
- ○ I'm not sure

Thank you for your interest, but only people over 18 years old who have MPN are eligible for this study.

Block 7

Block 1

What type of MPN do you have?

- ○ Polycythemia Vera (PV)
- ○ Essential Thrombocythemia (ET)
- ○ Myelofibrosis
- ○ Not sure

When were you diagnosed with your MPN?

- ○ Less than 1 year ago
- ○ 1-5 years ago
- ○ more than 5 years ago

What treatments are you taking for your MPN (check all that apply)

- ☐ aspirin

- [ ] therapeutic phlebotomy
- [ ] Interferon (pegasys)
- [ ] hydroxyurea (hydrea)
- [ ] anagrelide (agrylin)
- [ ] ruxolitinib (jakafi)
- [ ] fedratinib (inrebic)
- [ ] other

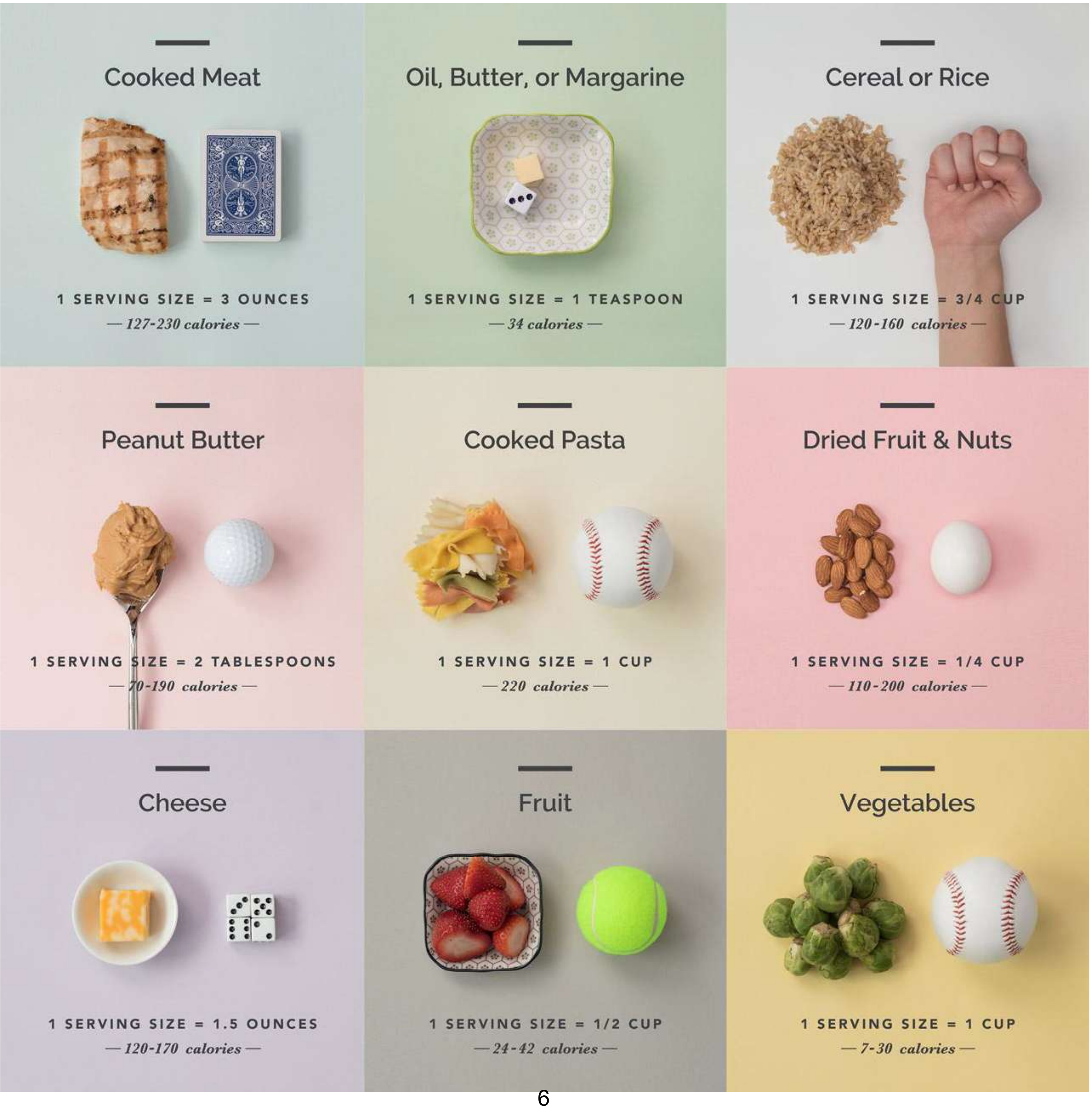

How much of each type of oil do you eat in a given day (including oil used for frying, salads, out-of-house meals, etc.)?

Number of TABLESPOONS (tbsp) per day

0	Olive Oil
0	Canola Oil
0	Vegetable or Corn Oil
0	Other Type of Oil

How many servings of vegetables do you consume per day? (1 serving : baseball size or 1 cup or 200 g [consider side dishes as half a serving])

▼

How many servings of fruit do you consume per day? (1 serving: 1 whole apple, orange, 1/2 cup cut fruit)

▼

How many servings of red meat, hamburger, or meat products (ham, sausage, etc.) do you consume per day? (1 serving: deck of cards or 3 oz or 100-150 g)

▼

How many servings of butter, margarine, or cream do you consume per day? (1 serving: 1 dice or 1 teaspoon or 12 g)

▼

How many sugar sweetened beverages do you drink per day? (such as regular soda, sweetened iced teas, lemonade, NOT diet)

How much alcohol do you drink per WEEK?

0 glasses of red wine

0 glasses of white wine

0 bottle/cans of beer

0 spirits (mixed drinks or hard alcohol)

0 other

How many servings of beans do you consume per week? (1 serving : fist size or 3/4 cup or 150 g)

How many servings of fish or shellfish do you consume per week? (1 serving: deck of cards or 3oz or 100-150 g of fish or 4-5 units or 1 cup or 200 g of shellfish)

How many times per week do you consume sweets or pastries, such as cakes, cookies, biscuits, or custard?

How many servings of nuts (including peanuts) do you consume per week? (1 serving: egg size or 1/2 cup or 30 g)

Which do you eat more of?

- ○ poultry/white meat

○ beef/hamburger, sausage, pork, veal

How many times per week do you eat dishes made with a sauce of onion, garlic, or vegetables simmered with olive oil (also known as sofrito)?

[▼]

How many times per week do you eat out at the following types of restaurants?

FAST FOOD RESTAURANTS<br>McDonalds, Burger Kings, Wendy's, Chick-fil-a, In-n-out	0
FAST CASUAL RESTAURANTS<br>Chipotle, Panera Bread, Boston Market	0
DELIVERY BASED<br>pizza deliveries, doordash, grubhub	0
HEALTH BASED<br>Tendergreens, Veggie Grill, Lyfe Kitchen, Sweet Green, Juice Bars	0
CASUAL DINING WITH SIT-DOWN AND TABLE SERVICE<br>Chili's, Applebee's, Olive Garden, Mom and Pop restaurants	0
FINE DINING RESTAURANTS	0
Total	0

Do you currently follow a specific diet?

○ Yes
○ No
○ Not Sure

Tell us a little about your diet

[ ]

Do you have any food allergies?

○ Yes

- No
- Maybe

Tell us about your food allergies

Block 2

Now we are going to ask you how you are feeling

NOTE: If your answer is 0, slide the bar forward then back to 0

What is your overall quality of life?

AS GOOD AS IT CAN BE						AS BAD AS IT CAN BE				
0	1	2	3	4	5	6	7	8	9	10

Please rate your fatigue (weariness, tiredness) with the one number that describes your worst level of fatigue DURING THE PAST 24 HOURS:

Absent						Worst Imaginable				
0	1	2	3	4	5	6	7	8	9	10

Filling up quickly when you eat (early satiety) DURING THE PAST 7 DAYS:

Absent						Worst Imaginable				
0	1	2	3	4	5	6	7	8	9	10

Abdominal Discomfort DURING THE PAST 7 DAYS:

Absent						Worst Imaginable				
0	1	2	3	4	5	6	7	8	9	10

Inactivity DURING THE PAST 7 DAYS:

		Absent					Worst Imaginable			
0	1	2	3	4	5	6	7	8	9	10

Problems with concentration (compared to before my MPN) DURING THE PAST 7 DAYS:

		Absent					Worst Imaginable			
0	1	2	3	4	5	6	7	8	9	10

Numbness/Tingling (in hands and feet) DURING THE PAST 7 DAYS:

		Absent					Worst Imaginable			
0	1	2	3	4	5	6	7	8	9	10

Night Sweats DURING THE PAST 7 DAYS:

		Absent					Worst Imaginable			
0	1	2	3	4	5	6	7	8	9	10

Itching (pruritis) DURING THE PAST 7 DAYS:

		Absent					Worst Imaginable			
0	1	2	3	4	5	6	7	8	9	10

Bone Pain (diffuse, not joint pain or arthritis) DURING THE PAST 7 DAYS:

		Absent					Worst Imaginable			
0	1	2	3	4	5	6	7	8	9	10

Fever (>100 F) DURING THE PAST 7 DAYS:

		Absent					Daily			
0	1	2	3	4	5	6	7	8	9	10

Unintentional weight loss IN THE LAST 6 MONTHS:

		Absent					Worst Imaginable			
0	1	2	3	4	5	6	7	8	9	10

Block 3

Are you willing to perform daily 1-2 minute symptom surveys prompted by a text or email?

- Yes
- No

Are you willing to participate in one on one dietician counseling sessions online?

- Yes
- No
- Maybe

Are you willing to change your diet to determine if this impacts your MPN symptoms?

- Yes
- No
- Maybe

Thank you for completing the survey. We will review your responses to determine if you are right for this study and get back to you within 2-3 days. Please provide us with your email address:

More info block

We'd be happy to give you more information on this study. Please enter your preferred contact information below (email or phone), also describe any specific questions you have. If you prefer you can also email us at wearempn@gmail.com

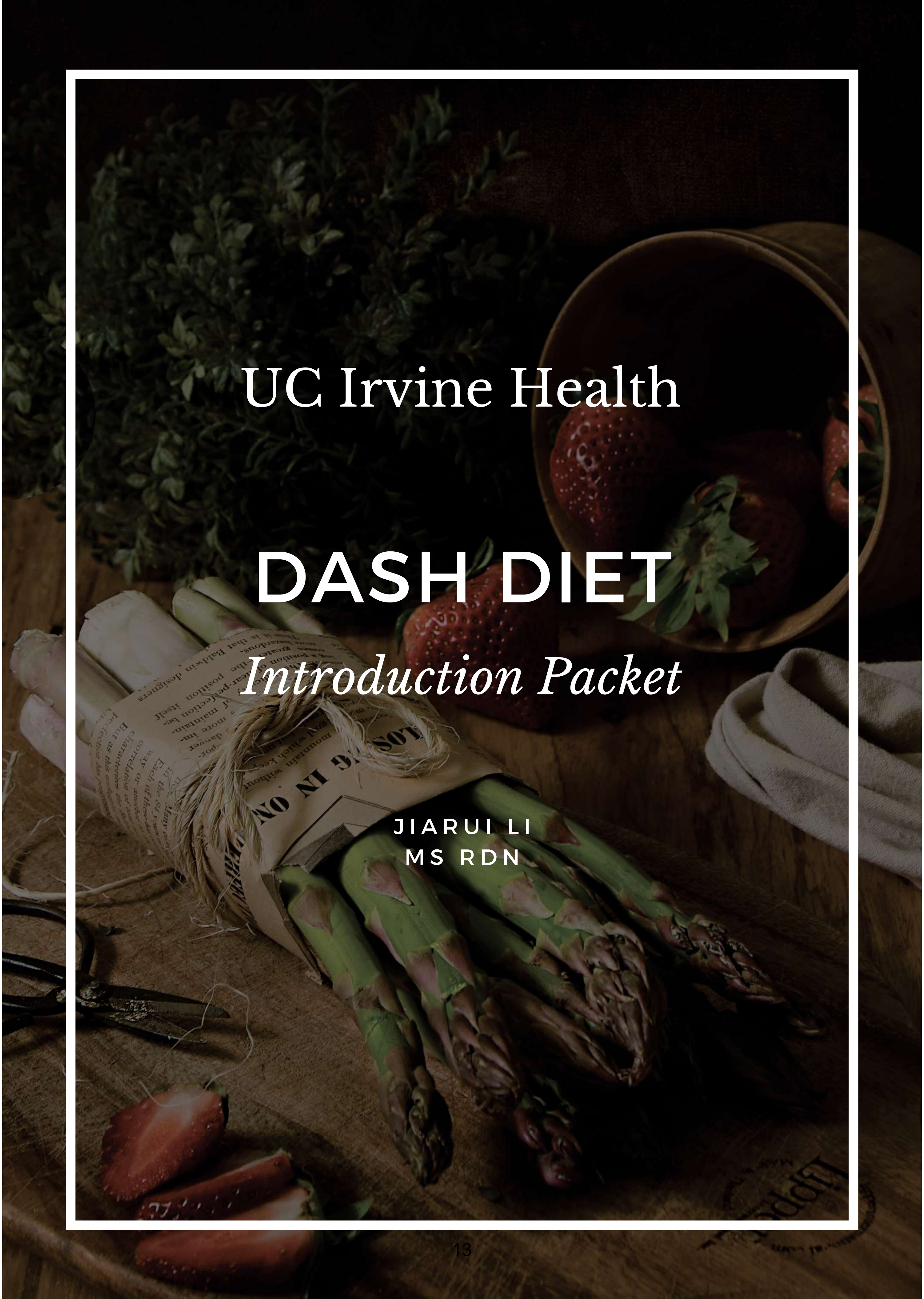

UC Irvine Health

DASH DIET

Introduction Packet

JIARUI LI
MS RDN

DASH
Overview

Dietary

Approaches to

Stop

Hypertension

THE DASH DIET IS DESIGNED TO TREAT AND PREVENT HIGH BLOOD PRESSURE (HYPERTENSION)

IT AIMS TO REDUCE SODIUM IN DIET AND TO PREVENT UNHEALTHY WEIGHT GAIN THROUGH OVER CONSUMPTION OF TOTAL CALORIES, SATURATED FATS, TRANS-FATS AND ADDED SUGARS.

IT IS AN APPROACH TO A HEALTHIER LIFESTYLE RATHER STRICTLY A 'DIET'

Why it matters to you?

- Chronic Inflammation is often associated with MPN.

- Our goal is to ease the inflammation burden through a healthy diet that has been shown to improve outcomes in previous studies.

- The DASH diet has many components which can be enhanced to improve inflammation.

Diet Components

Whole Grains
Low Fat dairy
Fruits
Vegetables
Beans
Legumes
Nuts
Lean Meats
Lower fats and oils
Minimum sweets and sugars

Lower Saturated Fats

Fats can help absorb vitamins and improve immune system. However, we should aim to limit saturated fat and trans fat which can increase risk of heart disease and inflammation.

- We recommend 1-2 tablespoons of unsaturated fats from oils such as olive oil, canola oil or vegetable oil for general cooking purposes.
- You may include salad dressing as a suitable alternative though you should opt for lower fat options.
- Limit or avoid high amounts of butter, margarine, palm oil, coconut oil, lard and other solid shortenings.

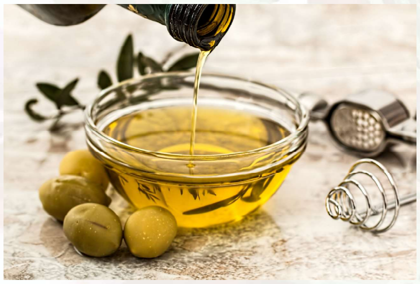

Poultry and Seafood

Lean poultry such as skinless chicken breast or turkey breast are excellent sources of iron and protein. Seafood can be excellent sources of protein as well and can include additional benefits from omega-3 fatty acids.

We recommend at most 3 poultry servings **per week.** 1 serving is about the size of cards.

If you choose to consume fish, opt for fatty fish such as salmon, herring and tuna. You can consume up to 1-2 servings **per week**.

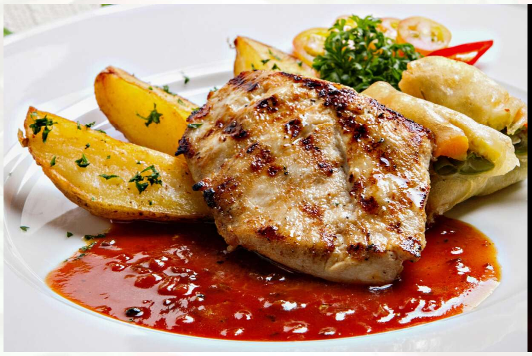

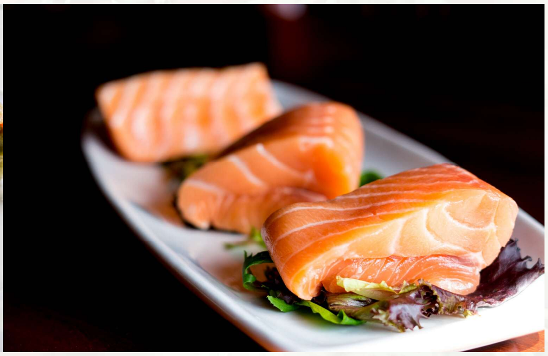

Meat

Meat is not an explicit part of the diet but can be consumed in small quantities. They can be a good source of protein and other nutrients such as zinc, Vitamin B12, phosphorus, iron and Vitamin K.

We recommend no more than 3-4 servings of lean red meats **per month.** 1 serving is about the size of a deck of cards.

We recommend trimming down visible fats and avoiding all processed meats

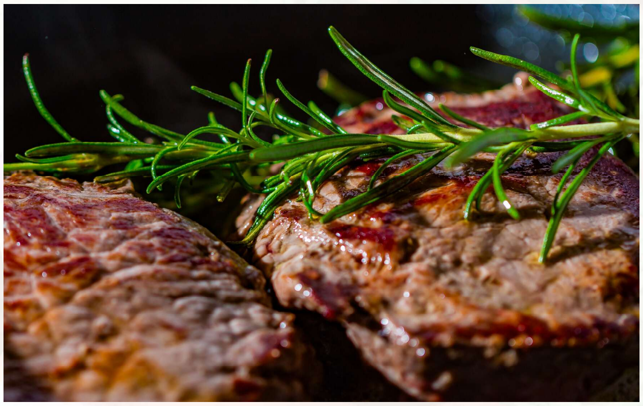

Eggs

Eggs are excellent protein sources and can provide significant amounts of vitamin A, B12, riboflavin, phosphorus and zinc.

We recommend no more than 4 eggs **per week.** Egg whites can be consumed without a limit.

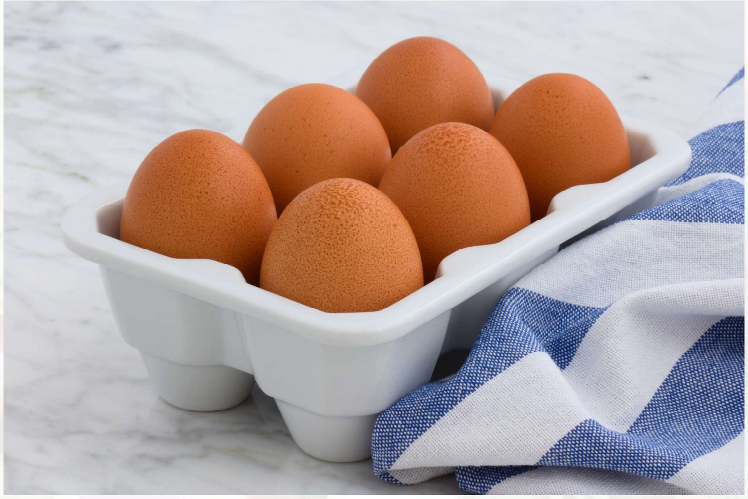

Low Fat Dairy

Dairy can be an easy way to get significant amounts of calcium, vitamin D and protein. It helps maintain bone density and reduce risk of fractures.

Fortified soy milk, almond milk, non-dairy yogurt and non-dairy cheese are suitable alternatives.

We recommend 1 cup of low-fat milk or yogurt, or 1 oz of low-fat cheese **per day.**

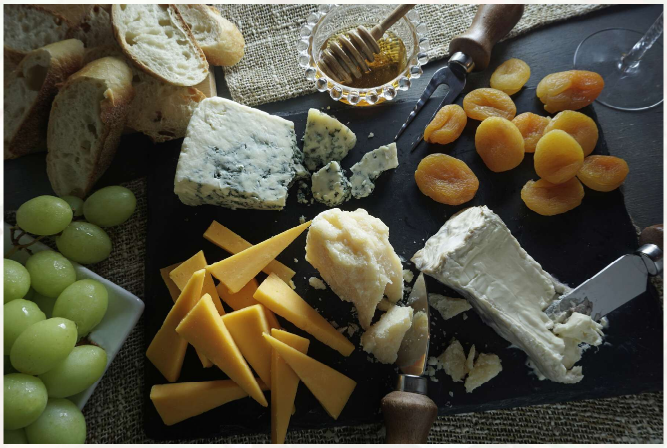

Beverages

We recommend that water be the main beverage of choice throughout the day. It can be enhanced with fruits, veggies or herbs. The typical recommendation of 8 glasses per day is suitable, with additional necessary if exercise is introduced.

We do not have any specific recommendations regarding coffee and other caffeinated drinks. While we don't generally recommend alcohol, men can consume up to 2 moderate drinks per day while women can consume up to 1.

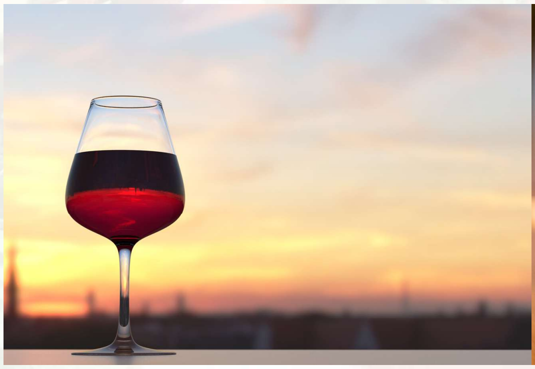

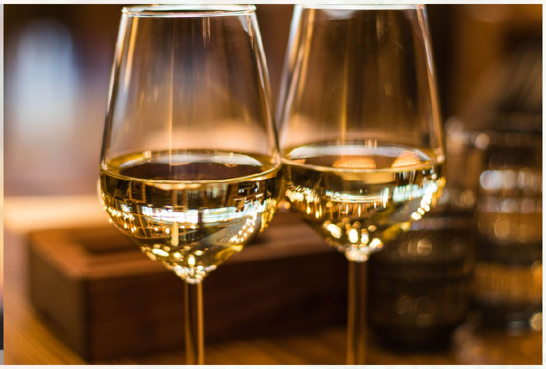

Legumes

Typically called beans, legumes can include soybeans, lentils, black beans, kidney beans, garbanzo, pinto and many others. They can be a significant source of protein, fiber and minerals.

We generally recommend up to 1 cup of <u>cooked</u> legumes **per day**.

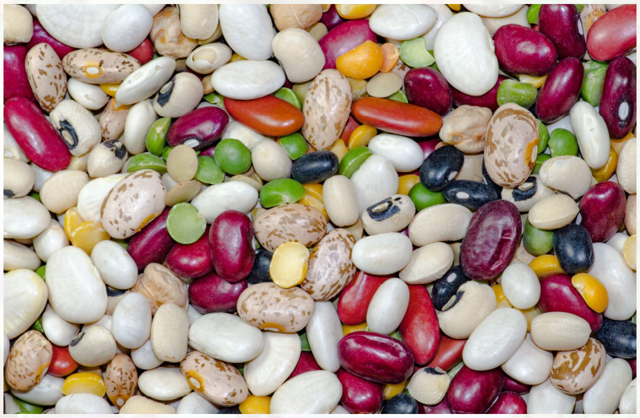

Whole Grains

These include unprocessed cereals such as: wheat, rice, barley, maize, rye, oats, quinoa, buckwheat.
They can also include flour based: bagels, sliced breads, flat breads, buns, rolls, pasta, crackers.

We generally recommend up to 2-3 servings of 100% whole grains **per day**.

1 serving size can be:

- 1 Slice of bread
- 1/2 large whole grain bun
- 1 small wheat grain roll
- 1/2 6-inch whole wheat pita bread
- 5 whole grain crackers
- 1/2 cup of cooked whole grain cereal
- 1/2 cup of whole wheat pasta or brown rice

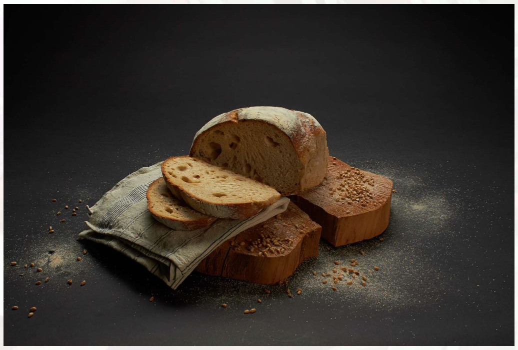

Starchy Vegetables

These are typically root vegetables such as potato, yam, beets, sweet potato, yacon, corn, sweet pea and others. They are nutritionally dense in fiber, antioxidants and a range of B-vitamins.

We generally recommend up to 2-3 servings of starchy vegetables **per day**.

1 serving size can be:

- 1/2 cup of cooked potatoes, sweet potatoes, corns and other starchy vegetables.

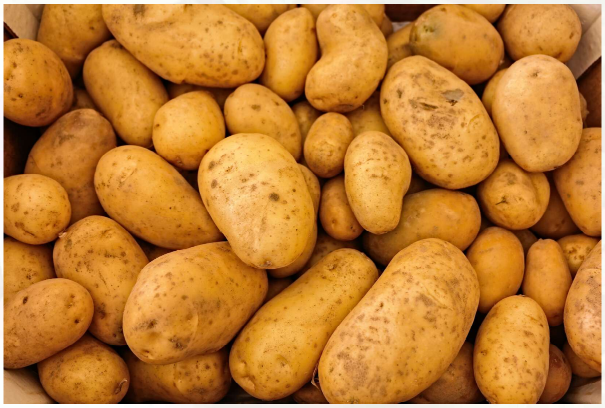

Non-Starchy Vegetables

These vegetables contain low amounts of starch and are typically very low in calories. They contain essential amounts of vitamins and minerals, and are also high in fiber and polyphenols.

They include typically what we generally think of vegetables e.g. broccoli, cauliflower, celery, carrots, tomatoes, cucumber, onions, green beans, salad greens and etc.

We generally recommend up to 4-8 servings of non-starchy vegetables **per day**.

1 serving size can be:

- 1/2 cup of cooked vegetables or 1 cup of raw vegetables.

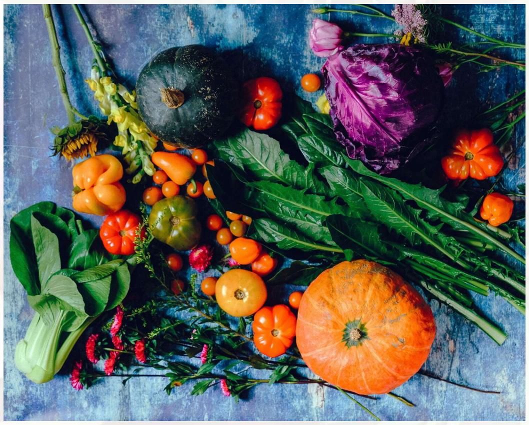

Fruits

They are the seed-bearing structures of flowering plants, and are typically high in fiber, vitamin C and water. Fruits can be fresh or canned. If you choose canned fruits, we recommend those canned in no-added sugar. Fruits canned in their own juices are fine.

We generally recommend up to 2-4 servings of fruits **per day**.

1 serving size can be:

- 1 small fruit or 1/4 cup of dried fruit.

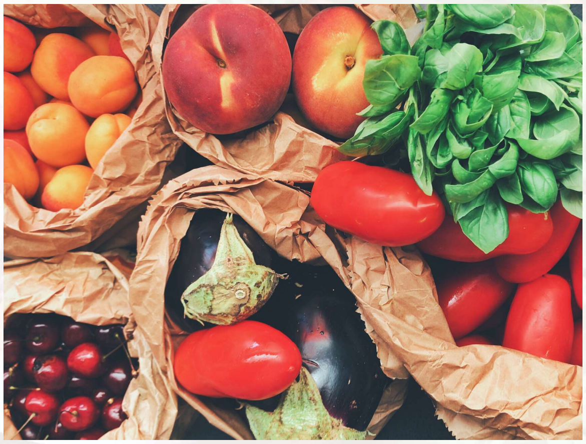

Sweets

We generally do not recommend frequent consumption of sweets, but you don't have to banish them entirely. In fact, certain sweets such as dark chocolate can be healthy when consumed in moderation. For sweets such as sugar, jelly, jam, sorbet or fruit juices such as lemonade, aim for less than 5 servings **per week.**

1 serving size can be:

- 1 tablespoon or sugar or syrup
- 1 table spoon of jelly or jam
- 1/2 cup of sorbet and other high sugar juices/drinks
- 1 cup of non-artificially sweetened fruit juice such as fresh orange juice, apple juice, pineapple juice and others.

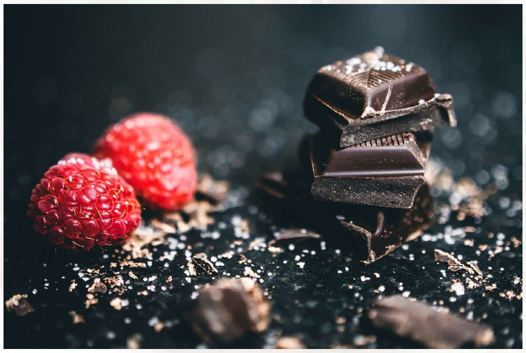

Sodium

Sodium can be present in many ways in our food. Sodium is an essential electrolyte that helps with fluid balance and muscle function. Its also an integral component of blood pressure maintenance. However, consuming high amounts of sodium may lead to hypertension.

Generally, we recommend you limit daily sodium from ALL foods to 2300 mg per day. In extreme circumstances, a limit of 1500 mg per day can be considered but in practicality it is very hard to achieve.

Please refer to the Sodium Guidelines packet to understand more about this topic.

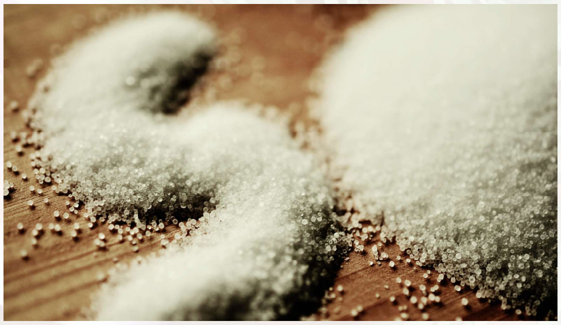

Cooking Techniques

- We generally recommend against frying or heavy oil use such as deep searing.
- For animal based foods such as poultry and meat: we recommend baking, sauteing, light stir-frying, roasting or grilling without skin.
- For fish, similar cooking techniques apply but you may keep the skin for cooking and consumption.
- For vegetables, we recommend baking, light-stir frying or steaming.

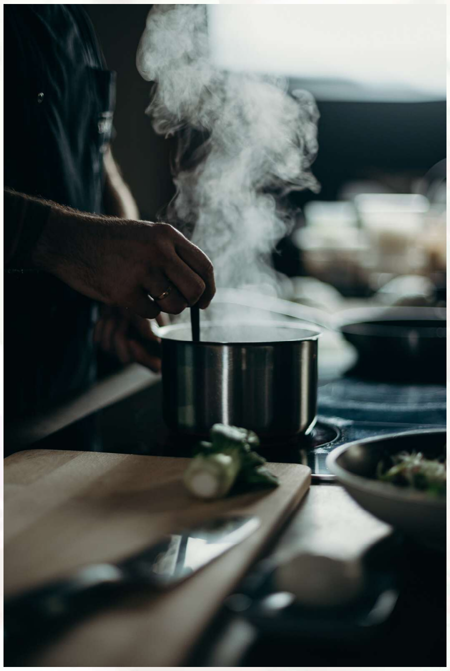

Miscellaneous

If there are any special dietary considerations or medically necessary information that you feel will conflict with this diet then please do not hesitate to contact us.

We have no recommendations on dietary supplementation including multivitamins. Many general dietary supplements may contain significant amounts of calories, fats and sugars. If you consume these frequently , then you may need to adjust your intake temporarily for this study.

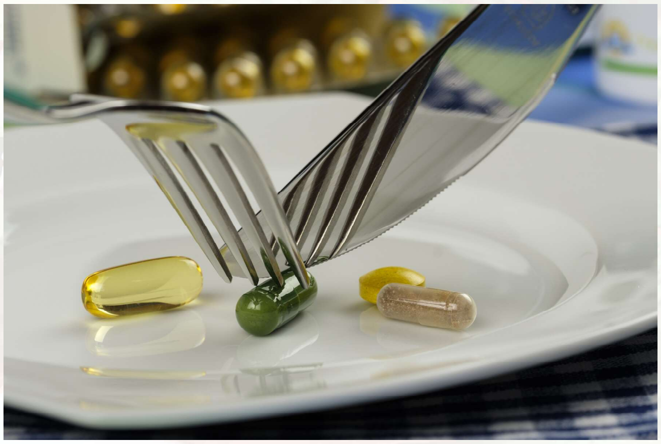

Physical Activity and Lifestyle

The 2018 Physical Activity Guidelines recommend that adults should move more and sit less throughout the day.

We recommend you to aim for at least 150 to 300 minutes of moderate intensity aerobic exercises. Alternatively, you may opt for 75 to 150 minutes of vigorous intensity aerobic exercises as well.

You should also aim to incorporate 2 or more days of muscle-building activities with high intensity lifting if appropriate.

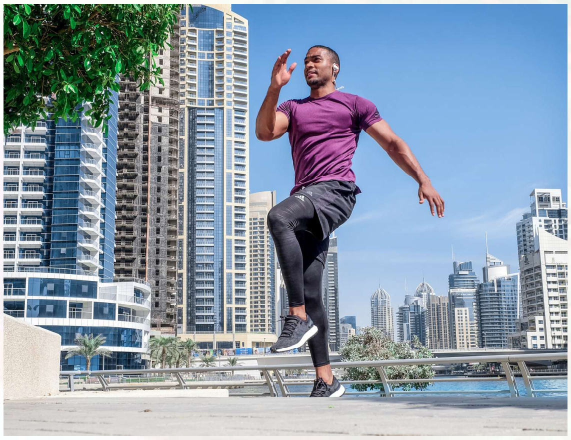

Conclusion

We hope this introductory packet can help you get started on this lifestyle challenge for the next few months while you are with us.

We encourage you to check out all available supplemental information we have included and if there are any questions please feel free to contact each of us when necessary.

Each of the dietitian sessions will focus on behavior counseling, goal setting, trouble shooting and general Q & A.

We will be following-up on each goal set in subsequent follow-up diet counseling sessions.

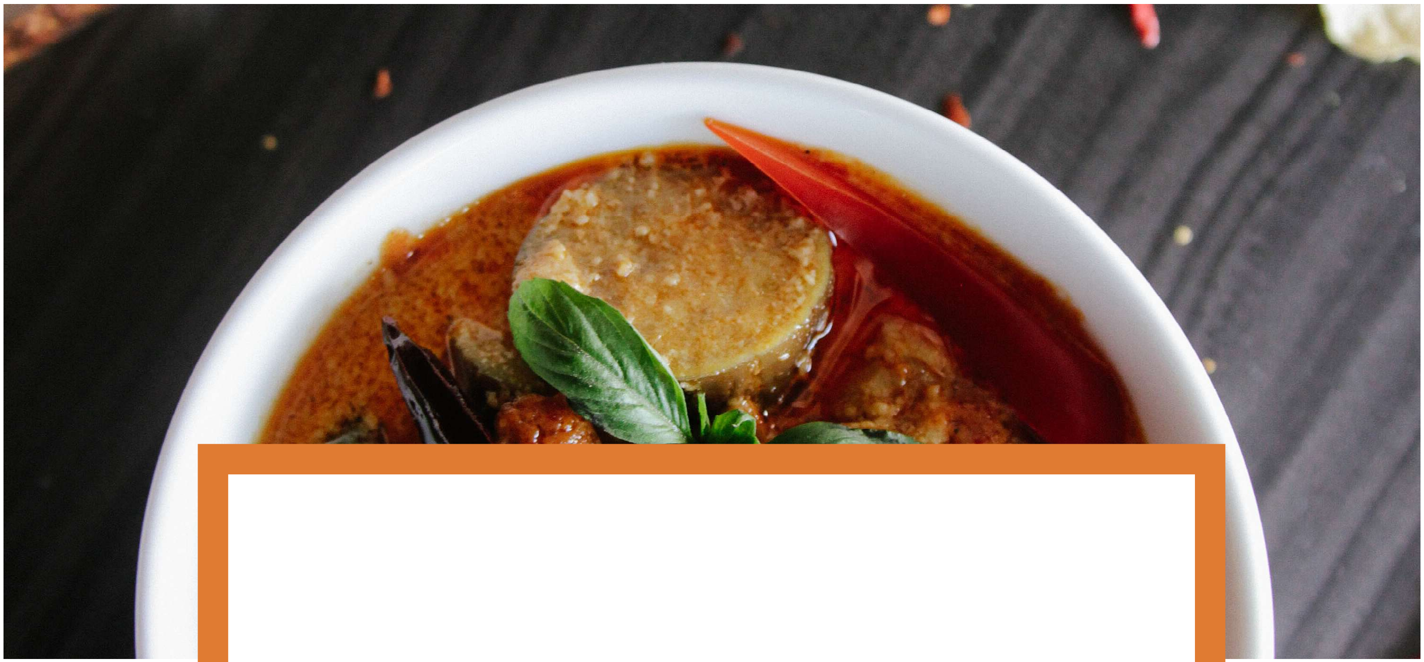

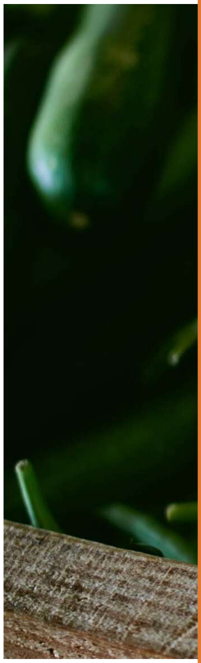

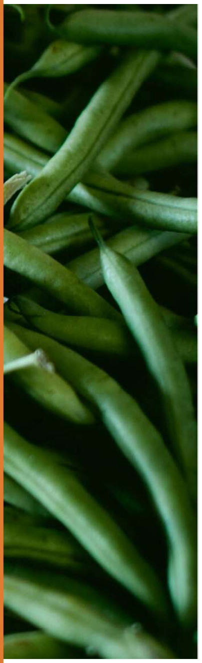

UC IRVINE HEALTH

MEDITERRANEAN DIET

JIARUI LI
MS RDN

INTRODUCTION PACKET

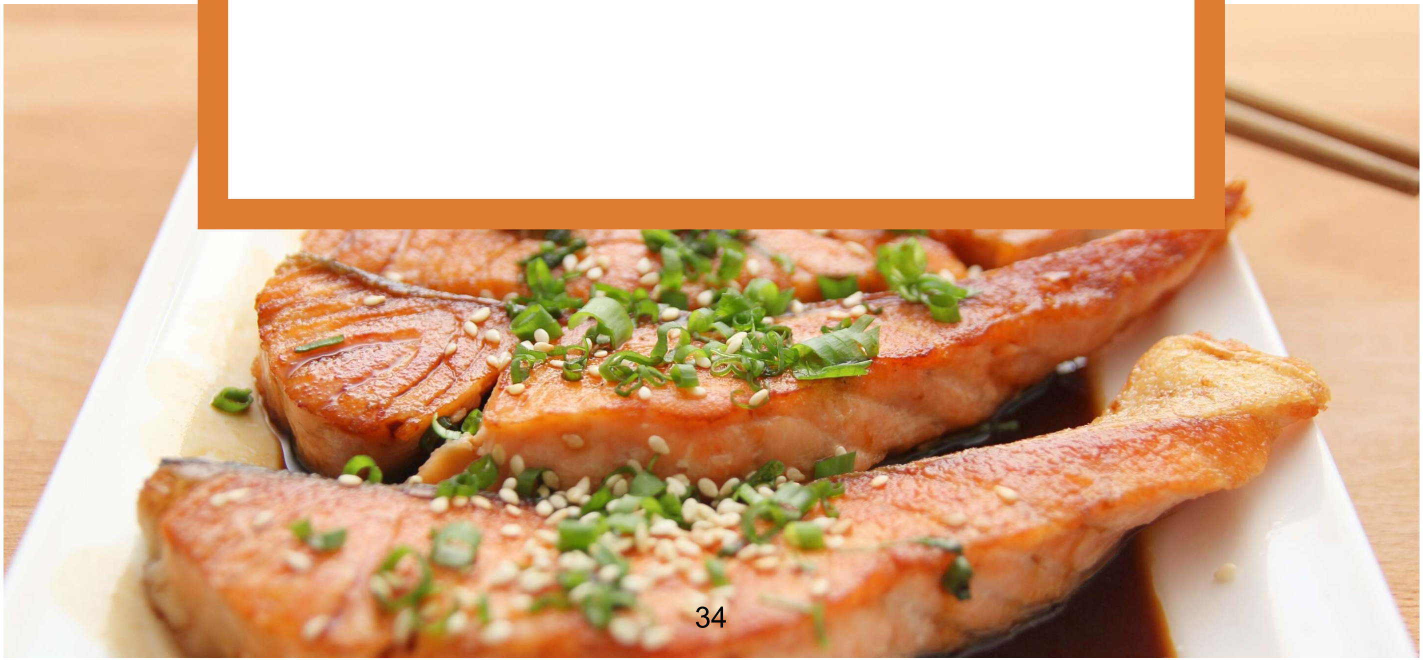

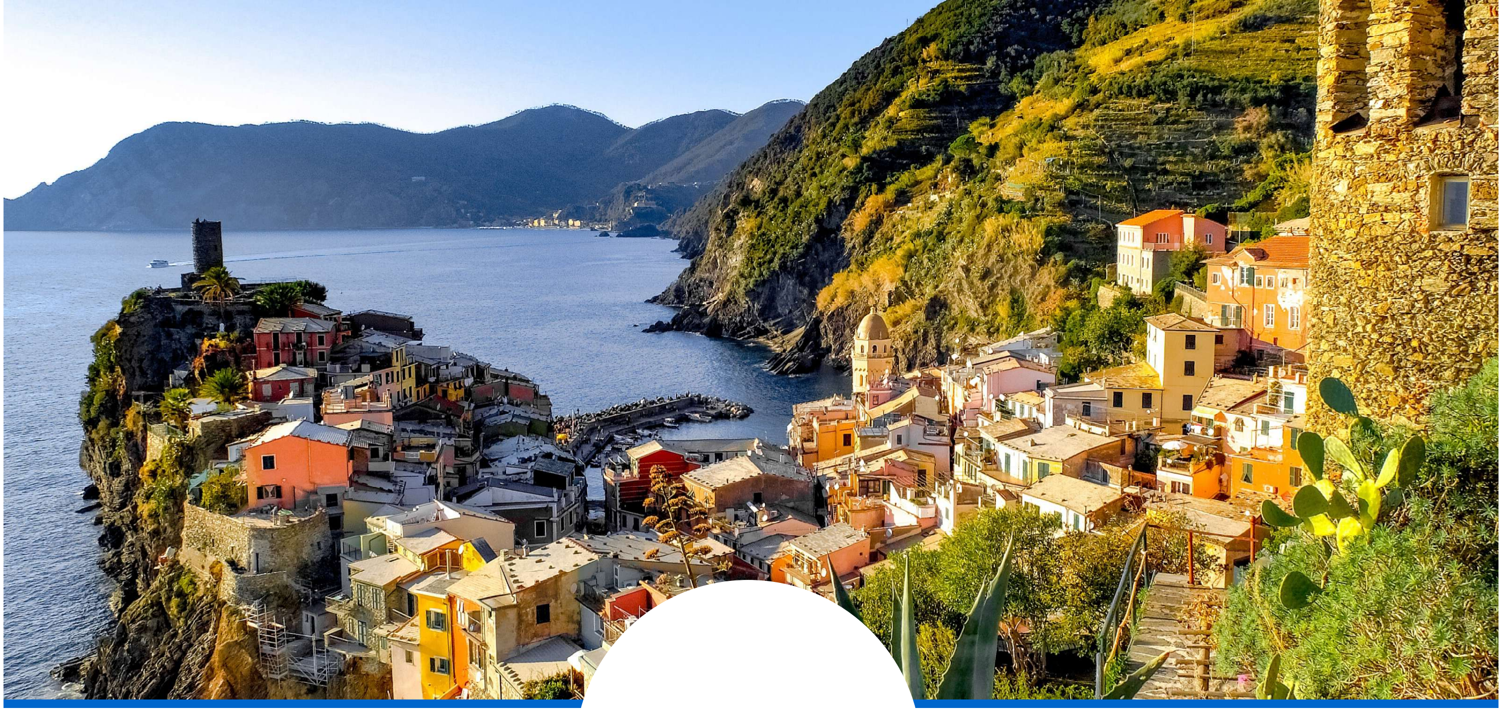

DIETARY OVERVIEW

The Mediterranean "diet" gained interest in the 1960s when studies revealed heart disease deaths were reduced in the Mediterranean Countries.

The dietary components itself is based on many of the region's traditional fruits, vegetables, beans, nuts, olive oil and seafood.

Its complexity is also well known for including red wine as an important part of its components.

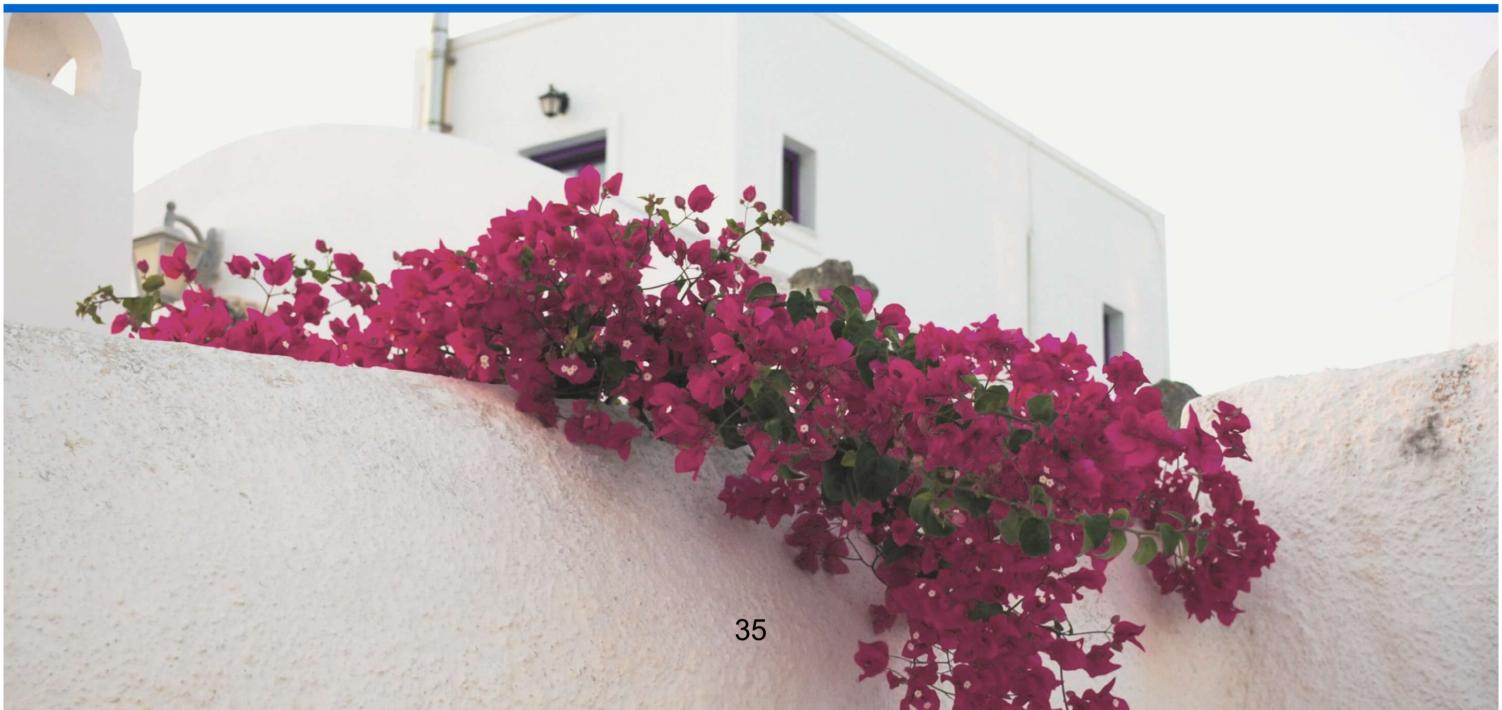

What does Research Say?

- PREDIMED Study is a multi-center trial in Spain in which participants were arranged into a control diet and a Mediterranean diet.
- Mediterranean diet group had significantly reduced risk of heart attacks.

- In the US a 2018 prospective cohort study examined 25,994 women in the 1993-1996 Women's Health Study.
- The group of women who consumed high quantities of Mediterranean foods had a 25% reduced risk of developing heart disease.

Why it matters to you?

- Chronic Inflammation is often associated with MPN.
- Our goal is to ease the inflammation burden through a healthy diet that has been shown to improve outcomes in previous studies.

- Mediterranean Diet pattern has been shown to have anti-inflammatory effects
- High adherence has been associated with lower biomarkers of inflammation (TNF-alpha, hs-CRP and PAI1 for example)

Diet Components

Whole Grains
Olive oil
Fruits
Vegetables
Beans
Legumes
Nuts
Herbs
Spices

What Components Separate the Mediterranean diet?

The Mediterranean diet has several unique features apart from the usual diet components listed before

- **The fat component is “healthy”** – addition of extra-virgin olive oil, nuts and seeds.
- **Omega-3 fatty acid rich fish** are consistent parts of diet
- **Wine** with daily meals
- **Sofrito** sauce for cooking and seasoning
- Liberal use of **herbs and spices**

Healthy Fats

Fats should be mostly unsaturated and little saturated. No trans-fat should be used.

- **Extra-Virgin Olive Oil** – Contains high amounts of antioxidants and polyphenols to combat inflammation
- **Avocados -** It is actually a fruit, but 75% unsaturated fat. It is very rich in vitamins B, E and minerals
- **Salad Dressings -** Aim to use light salad dressing with little or no saturated fat. Ideally aim for lower sodium alternatives as well.

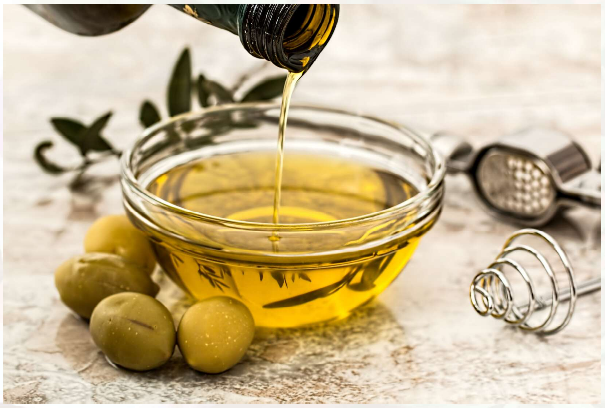

Healthy Fats

We recommend:

- 1-2 tablespoons of extra-virgin olive oil for cooking and salads **per day**
- Up to 1/2 or 1 medium avocado **per day**
- 1-2 tbsp of light salad dressing **per day**

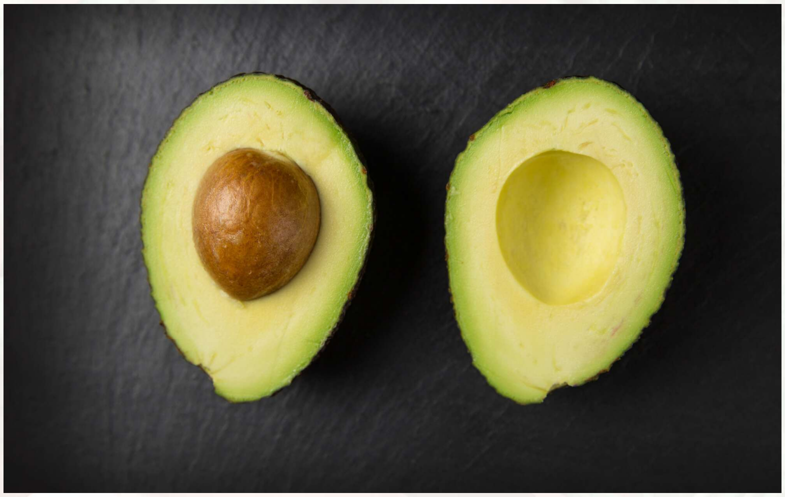

Nuts and Seeds

Nuts and seeds are high in unsaturated fat and has been liked to improving cholesterol.

- They contain high amounts of omega 3 fatty acids, fiber, Vitamin E and sterols

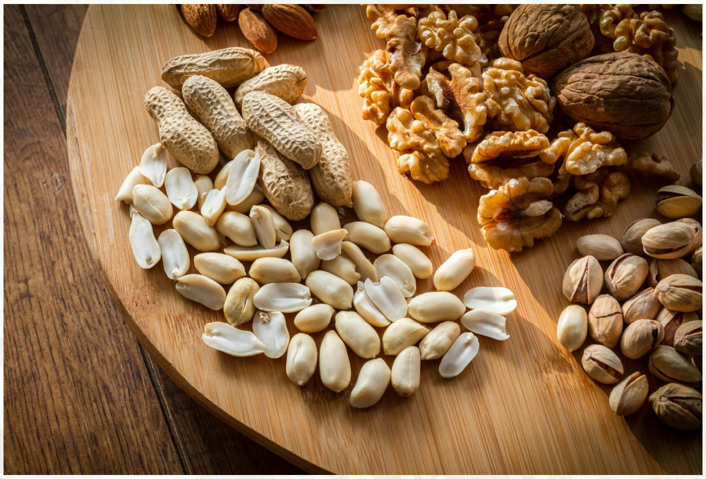

Nuts and Seeds

All seed varieties are fine (e.g. chia, flaxseeds, hemp, pumpkin, sesame, sunflower and etc).

- We recommend no more than 1/4 - 1/2 cups of nuts and seeds **per day**.

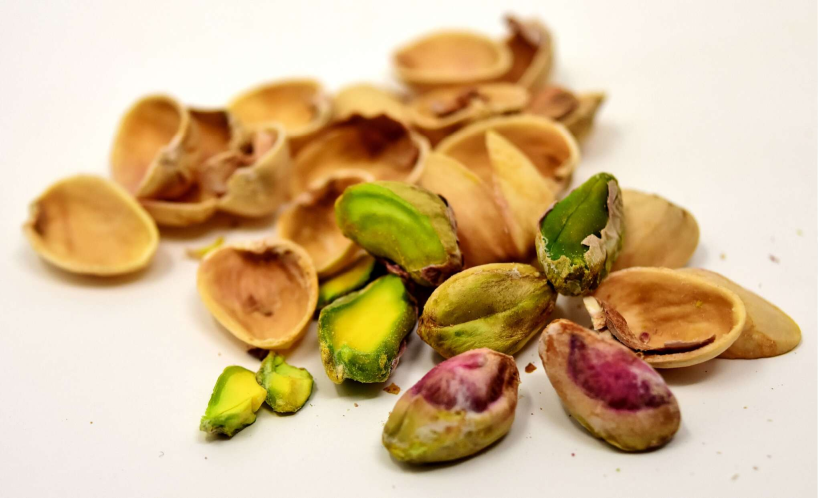

Seafood

Seafood is an excellent source of protein. Fatty fish are good sources of omega-3 fatty acids, which has been shown to reduce risk of heart disease.

They include: Salmon, Mackerel, Herring, Trout, Sardine, and albacore tuna.

We recommend 1-2 servings of fatty fish **per week.** 1 serving is about the size of a deck of cards.

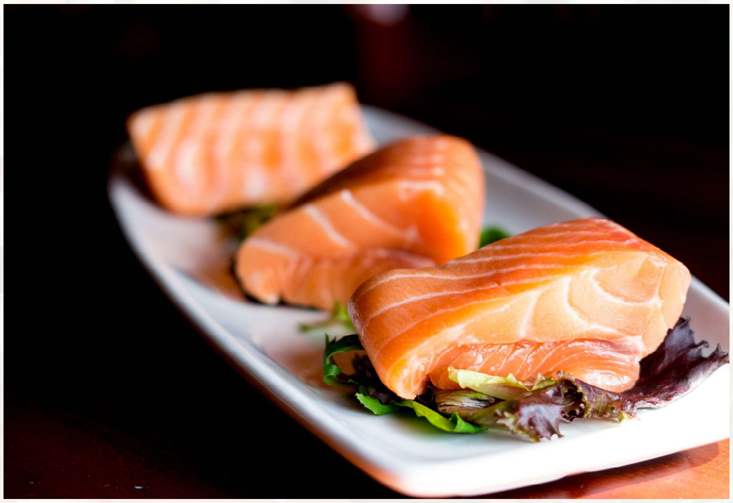

Poultry

Lean poultry such as skinless chicken breast or turkey breast are excellent sources of iron and protein.

We recommend at most 3 servings **per week.** 1 serving is about the size of a deck of cards.

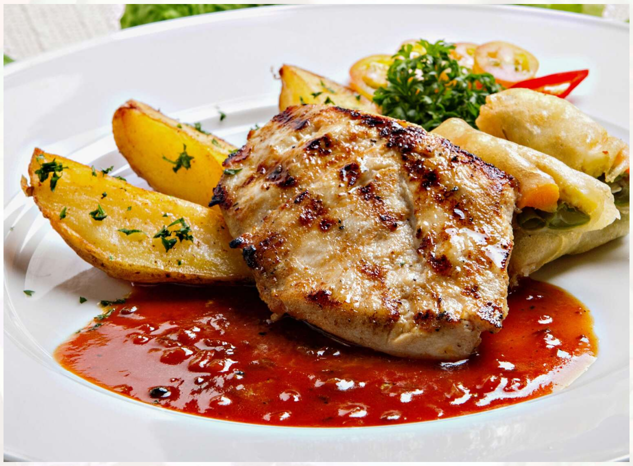

Meat

Meat is not an explicit part of the diet but can be consumed in small quantities. They can be a good source of protein and other nutrients such as zinc, Vitamin B12, phosphorus, iron and Vitamin K.

We recommend no more than 3-4 servings of lean red meats **per month.** 1 serving is about the size of a deck of cards.

We recommend trimming down visible fats and avoiding all processed meats

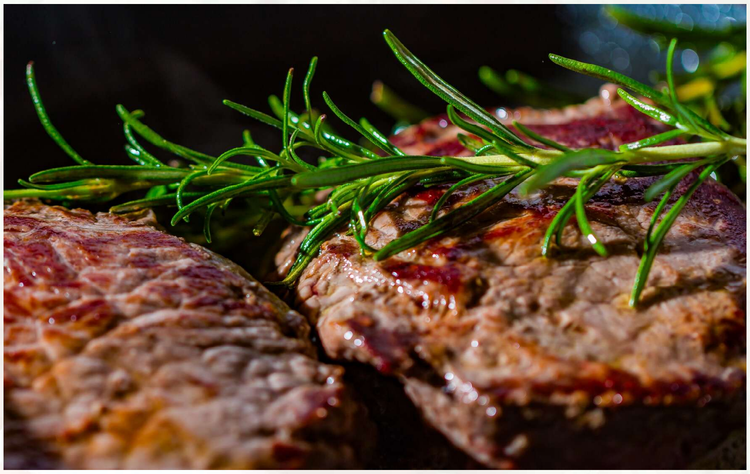

Eggs

Eggs are excellent protein sources and can provide significant amounts of vitamin A, B12, riboflavin, phosphorus and zinc.

We recommend no more than 4 eggs **per week.** Egg whites can be consumed without a limit.

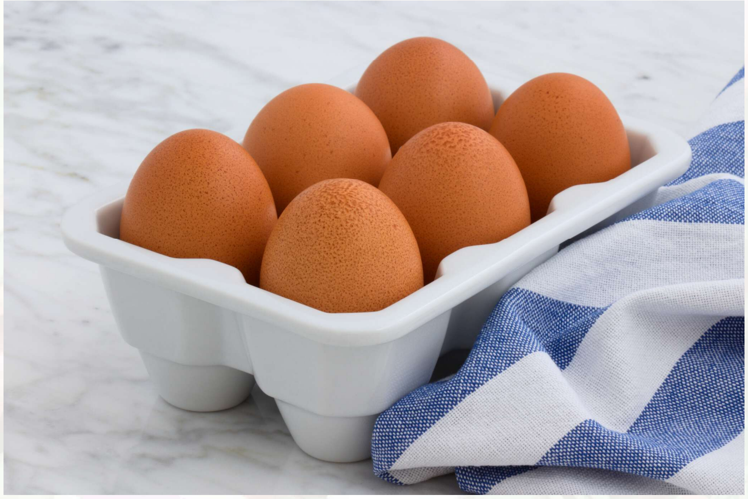

Low Fat Dairy

Dairy can be an easy way to get significant amounts of calcium, vitamin D and protein. It helps maintain bone density and reduce risk of fractures.

Fortified soy milk, almond milk, non-dairy yogurt and non-dairy cheese are suitable alternatives.

We recommend 1 cup of low-fat milk or yogurt, or 1 oz of low-fat cheese **per day.**

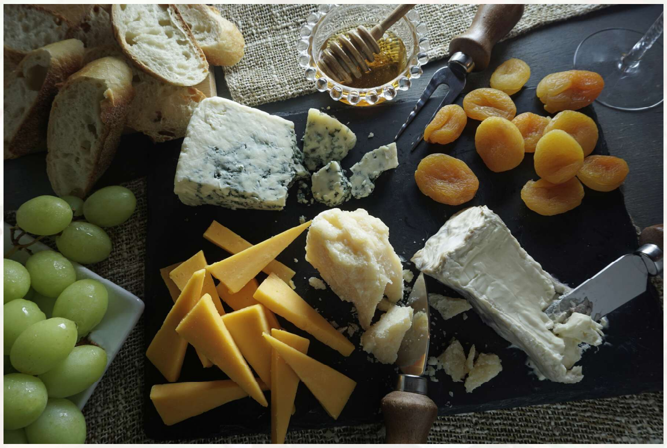

Beverages

We recommend that water be the main beverage of choice throughout the day. It may be enhanced with fruit, veggies or herbs. The typical recommendation of 8 glasses per day is suitable, with additional necessary if exercise is introduced.

We also recommend you to consume moderate amounts of red wine with meals:

- Men can consume up to **2 glasses a day**
- Women can consume up to **1 glass a day**

We do not recommend the consumption of artificially or sugar-sweetened beverages such as sodas, juices or other enhanced beverages.

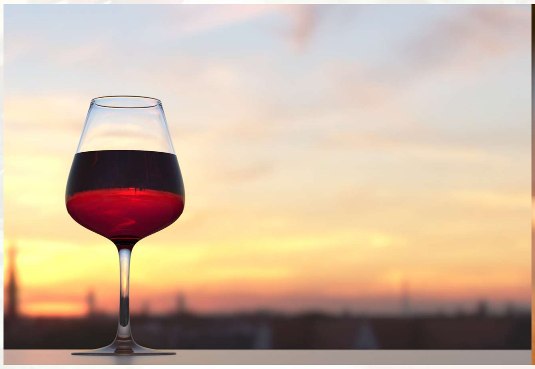

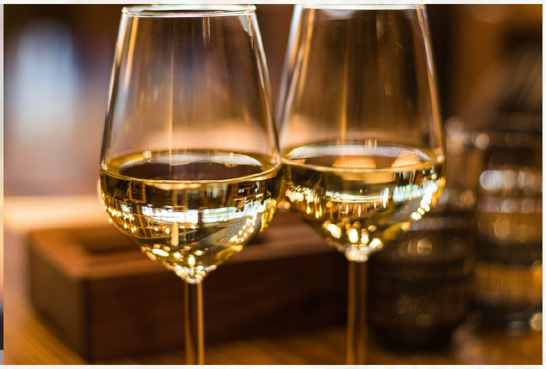

Legumes

Typically called beans, legumes can include soybeans, lentils, black beans, kidney beans, garbanzo, pinto and many others. They can be significant source of protein, fiber and minerals.

We generally recommend up to 1 cup of <u>cooked</u> legumes **per day**.

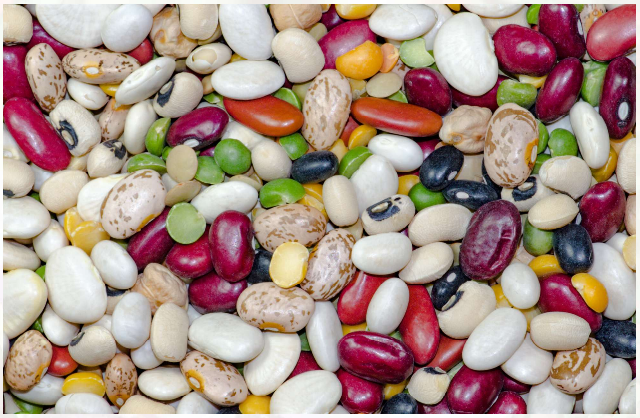

Whole Grains

These include unprocessed cereals such as: wheat, rice, barley, maize, rye, oats, quinoa, buckwheat.
They can also include flour based: bagels, sliced breads, flat breads, buns, rolls, pasta, crackers.

We generally recommend up to 2-3 servings of 100% whole grains **per day**.

1 serving size can be:

- 1 Slice of bread
- 1/2 large whole grain bun
- 1 small wheat grain roll
- 1/2 6-inch whole wheat pita bread
- 5 whole grain crackers
- 1/2 cup of cooked whole grain cereal
- 1/2 cup of whole wheat pasta or brown rice

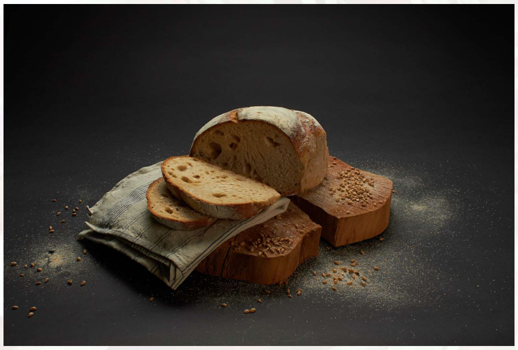

Starchy Vegetables

These are typically root vegetables such as potato, yam, beets, sweet potato, yacon, corn, sweet pea and others. They are nutritionally dense in fiber, antioxidants and a range of B-vitamins.

We generally recommend up to 2-3 servings of starchy vegetables **per day**.

1 serving size can be:

- 1/2 cup of cooked potatoes, sweet potatoes, corns and other starchy vegetables.

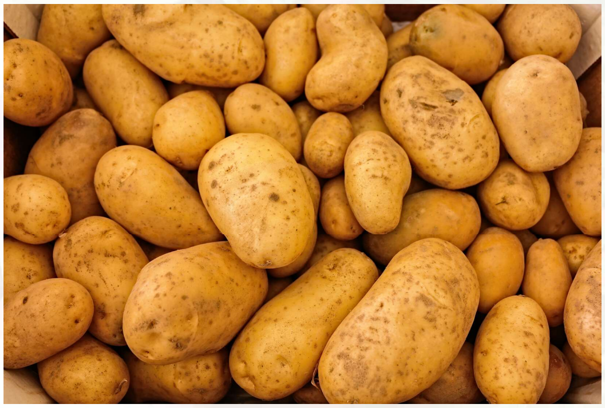

Non-Starchy Vegetables

These vegetables contain low amounts of starch and are typically very low in calories. They contain essential amounts of vitamins and minerals, and are also high in fiber and polyphenols.

They include typically what we generally think of vegetables e.g. broccoli, cauliflower, celery, carrots, tomatoes, cucumber, onions, green beans, salad greens and etc.

We generally recommend up to 4-8 servings of non-starchy vegetables **per day**.

1 serving size can be:

- 1/2 cup of cooked vegetables or 1 cup of raw vegetables.

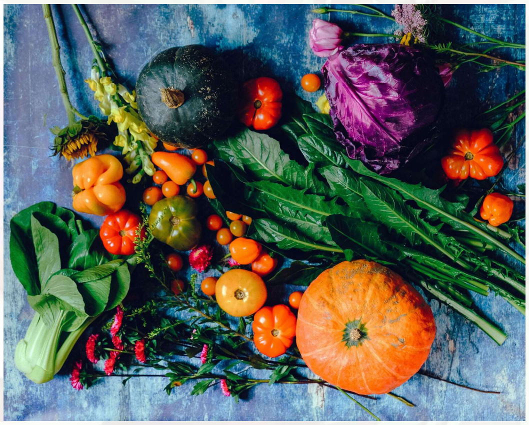

Fruits

They are the seed-bearing structure of flowering plants, and are typically high in fiber, vitamin C and water. Fruits can be fresh or canned. If you choose canned fruits, we recommend those canned in no-added sugar. Fruits canned in their own juices are fine.

We generally recommend up to 2-4 servings of fruits **per day**.

1 serving size can be:

- 1 small fruit or 1/4 cup of dried fruit.

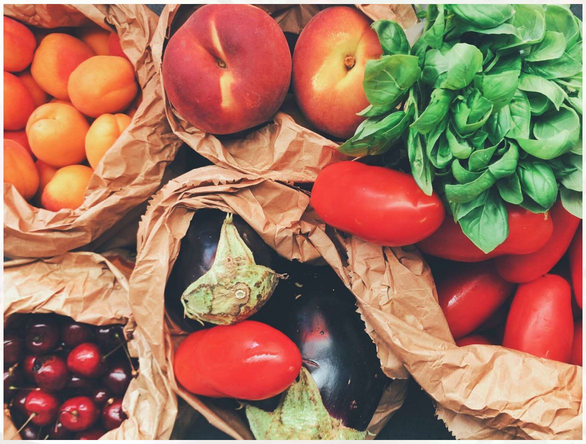

Cooking Techniques

- We generally recommend against frying or heavy oil use such as deep searing.
- For animal based foods such as poultry and meat: we recommend baking, sauteing, light stir-frying, roasting or grilling without skin.
- For fish, similar cooking techniques apply but you may keep the skin for cooking and consumption.
- For vegetables, we recommend baking, light-stir frying or steaming.

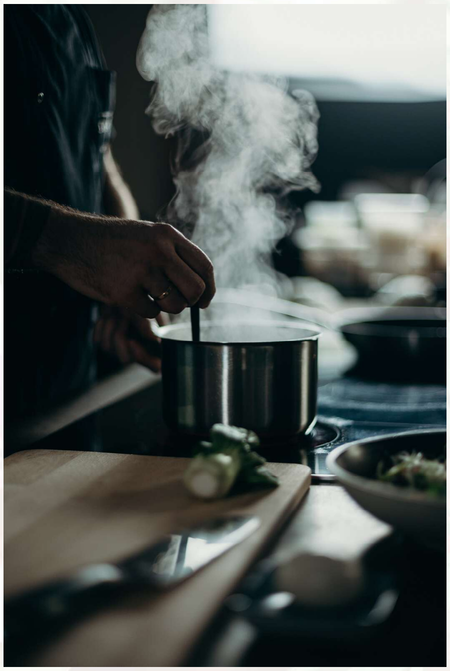

Mediterranean Herbs and Spices

Herbs and Spices can often add additional flavor to your foods without the need for sodium. They are fantastic salt alternatives!

These can be: parsley, oregano, saffron, rosemary, sage, cilantro, basil, bay leaf, pepper, cloves, coriander, cumin, garlic, mint and many more.

We generally recommend you to use them as tolerated during cooking and meal preparation. Make sure to check our supplemental herb and spices guide!

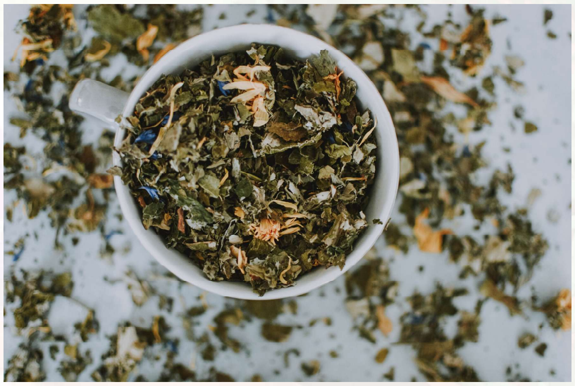

Sofrito

This is a Mediterranean dish that consists of chopping up garlic, onion, pepper, tomatoes, celery and other greens. It is further seasoned then lightly topped off with olive oil.

It maybe served as a side to meals or as a marinade for fish, poultry or meat.

Make sure to check out our supplemental sofrito guide.

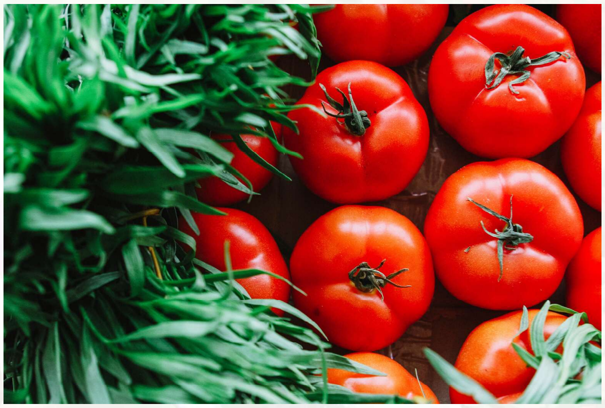

Miscellaneous

If there are any special dietary considerations or medically necessary information that you feel will conflict with this diet then please do not hesitate to contact us.

We have no recommendations on dietary supplementation including multivitamins. Many general dietary supplements may contain significant amounts of calories, fats and sugars. If you consume these frequently , then you may need to adjust your intake temporarily for this study.

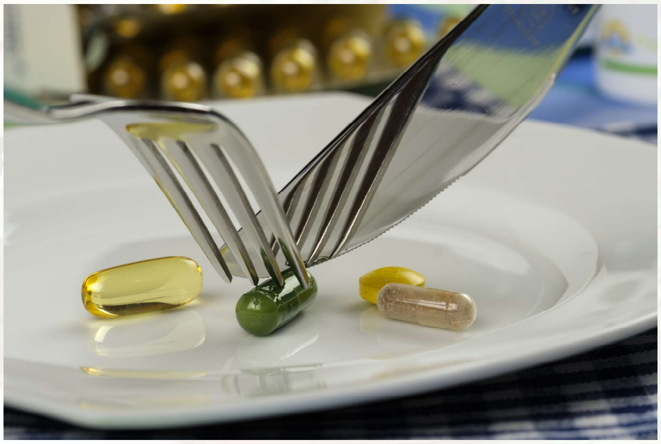

Physical Activity and Lifestyle

The 2018 Physical Activity Guidelines recommend that adults should move more and sit less throughout the day.

We recommend you to aim for at least 150 to 300 minutes of moderate intensity aerobic exercises. Alternatively, you may opt for 75 to 150 minutes of vigorous intensity aerobic exercises as well.

You should also aim to incorporate 2 or more days of muscle-building activities with high intensity lifting if appropriate.

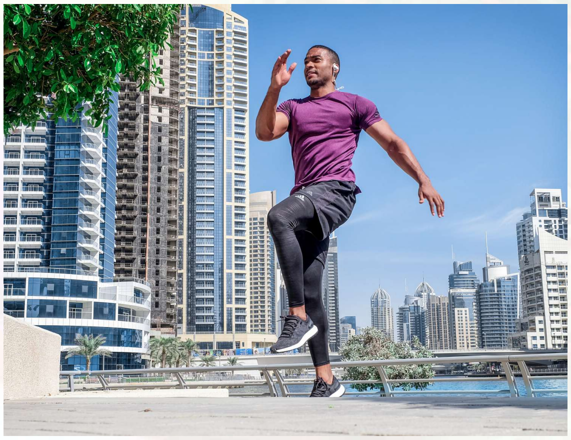

Conclusion

We hope this introductory packet can help you get started on this lifestyle challenge for the next few months while you are with us.

We encourage you to check out all available supplemental information we have included and if there are any questions please feel free to contact each of us when necessary.

Each of the dietitian sessions will focus on behavior counseling, goal setting, trouble shooting and general Q & A.

We will be following-up on each goal set in subsequent follow-up diet counseling sessions.
